# ATRX silences *Cartpt* expression in osteoblastic cells during skeletal development

**DOI:** 10.1172/JCI163587

**Published:** 2025-01-02

**Authors:** Yi-Ting Chen, Ming-Ming Jiang, Carolina Leynes, Mary Adeyeye, Camilla F. Majano, Barakat Ibrahim, Urszula Polak, George Hung, Zixue Jin, Denise G. Lanza, Lan Liao, Brian Dawson, Yuqing Chen-Evenson, Oscar E. Ruiz, Richard J. Gibbons, Jason D. Heaney, Yangjin Bae, Brendan Lee

**Affiliations:** 1 Integrative Molecular and Biomedical Sciences Program and; 2 Department of Molecular and Human Genetics, Baylor College of Medicine, Houston, Texas, USA.; 3 Genetics and Epigenetics Program, University of Texas Health Science Center at Houston, Houston, Texas, USA.; 4 Department of BioSciences, Rice University, Houston, Texas, USA.; 5 Department of Molecular and Cellular Biology, Baylor College of Medicine, Houston, Texas, USA.; 6 MRC Molecular Hematology Unit, Weatherall Institute of Molecular Medicine, University of Oxford, John Radcliffe Hospital, Oxford, United Kingdom.

**Keywords:** Bone biology, Bone development

## Abstract

ATP-dependent chromatin remodeling protein ATRX is an essential regulator involved in maintenance of DNA structure and chromatin state and regulation of gene expression during development. *ATRX* was originally identified as the monogenic cause of X-linked α-thalassemia mental retardation (ATR-X) syndrome. Affected individuals display a variety of developmental abnormalities and skeletal deformities. Studies from others investigated the role of ATRX in skeletal development by tissue-specific *Atrx* knockout. However, the impact of ATRX during early skeletal development has not been examined. Using preosteoblast-specific *Atrx* conditional knockout mice, we observed increased trabecular bone mass and decreased osteoclast number in bone. In vitro coculture of *Atrx* conditional knockout bone marrow stromal cells (BMSCs) with WT splenocytes showed impaired osteoclast differentiation. Additionally, *Atrx* deletion was associated with decreased receptor activator of nuclear factor κ-B ligand (*Rankl*)/ osteoprotegerin (*Opg*) expression ratio in BMSCs. Notably, *Atrx*-deficient osteolineage cells expressed high levels of the neuropeptide cocaine- and amphetamine-regulated transcript prepropeptide (*Cartpt*). Mechanistically, ATRX suppresses *Cartpt* transcription by binding to the promoter, which is otherwise poised for *Cartpt* expression by RUNX2 binding to the distal enhancer. Finally, *Cartpt* silencing in *Atrx* conditional knockout BMSCs rescued the molecular phenotype by increasing the *Rankl*/*Opg* expression ratio. Together, our data show a potent repressor function of ATRX in restricting *Cartpt* expression during skeletal development.

## Abstract 2

ATRX deficiency in early osteoblastic lineage increases bone mass due to loss of physiological transcriptional silencing of *Cartpt* resulting in decreased RANKL/OPG-mediated osteoclastogenesis.

## Graphical abstract

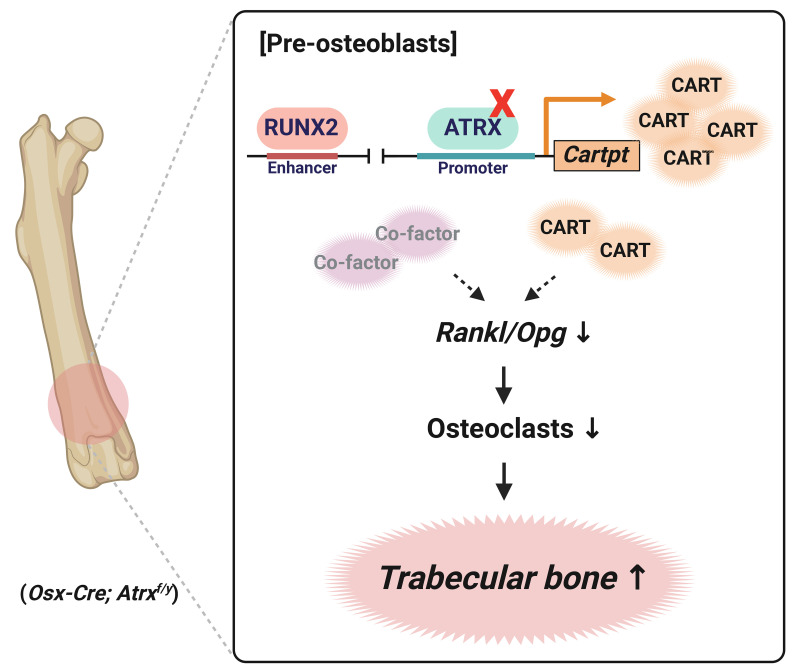

## Introduction

ATRX protein is a chromatin remodeler involved in multiple cellular processes including maintenance of chromatin status and DNA structure and regulation of gene expression (1–3). Playing essential roles in diverse regulatory processes, *ATRX* mutations are linked to 2 major disease conditions: germline mutations are responsible for X-linked α-thalassemia mental retardation (ATR-X) syndrome, while somatic mutations are prevalent in a variety of cancers such as brain cancer, pancreatic cancer, soft tissue sarcoma, and osteosarcoma (4–7).

ATRX contains 2 highly conserved domains: ADD (ATRX-DNMT3- DNMT3L) and ATPase/helicase domains. The N-terminal ADD domain contains GATA-like zinc finger, supporting a function in DNA binding (8, 9). This domain targets ATRX to heterochromatin regions by recognizing histone marks H3K9me3/H3K4me0 (10, 11). In line with this feature, ATRX was identified to cooccupy with other chromatin factors at 3′ exons of zinc-finger genes to maintain genomic stability by preservation of H3K9me3 (12). The C-terminal ATPase/helicase domain, on the other hand, hydrolyzes ATP to remodel chromatin or to mediate chromatin accessibility (13). By interacting with death-domain associated protein (DAXX), a ATRX-DAXX histone chaperone complex deposits the histone variant H3.3 at telomeric/pericentromeric, repetitive DNA, and euchromatic regions (14–16). Although the precise function of the H3.3 deposition remains unclear, several studies have demonstrated that this histone incorporation could be one of the mechanisms for protecting cells from G-quadruplex–induced DNA replication stress and for maintaining genomic stability (1, 12).

As one of the SWItch/sucrose non-fermentable (SWI/SNF) family of chromatin remodelers, ATRX has been reported to regulate gene expression. ATRX negatively modulates histone variant microH2A deposition, which is associated with inactivated transcription, at the α-globin gene cluster (3). In addition, a study by Levy et al. showed that ATRX promoted activation of *Nlgn4* expression by incorporation of histone variant H3.3 at the gene body to facilitate transcription elongation through guanine-rich coding regions (2). In the absence of ATRX, the inability to transcribe the α-globin genes cluster and NLGN4 gene could contribute to reducing α-globin expression and deficits in neuronal function, which are primary features of ATR-X syndrome patients (2, 3). Together, these findings suggest a critical role of ATRX in regulation of gene expression.

ATR-X syndrome is a rare genetic disorder caused by mutations in the X-linked *ATRX* gene (17). Hemizygous male ATR-X syndrome patients display a variety of developmental abnormalities, including cognitive impairment, α-thalassemia, and skeletal deformities (18, 19). To investigate the role of ATRX in skeletal development, 3 mouse models of cell-type–specific *Atrx* deletion using *Col2a1-*, *Prx1-*, and *Col1a1-Cre* have been studied in the past, which specifically deleted *Atrx* at the cartilage, forelimb mesenchyme, and bone-forming osteoblasts, respectively (20–22). Although *Atrx* deletion in chondrocytes had minimal effects during skeletal development, loss of *Atrx* in forelimb mesenchyme and osteoblasts caused brachydactyly and minor dwarfism, respectively (20–22). These findings suggest that *Atrx* deficiency in osteoblastic lineage, instead of chondrocytes, may lead to abnormal bone remodeling. However, the biological roles of cell-type–specific loss of ATRX function in osteoblastic lineage on bone formation and resorption were not further studied in these models.

Cocaine and amphetamine-regulated transcript (CART) peptides are encoded by the highly conserved *CARTPT* gene (23). These 116 amino acids–long CART prepropeptides are cleaved and processed through the regulated secretory pathway in Golgi into 2 bioactive peptides, CART 42–89 and 49–89 (24), which are homologous to CART 55–102 and 62–102 in rats and mice (25). CART peptides have been found in the central and peripheral nervous systems (26, 27). Moreover, *CARTPT* is expressed in the endocrine cells in the pancreatic islets (28), gastrointestinal tract mucosa (29), and adrenal medulla (30). However, bone cells do not express *CARTPT* in the skeleton (31–33).

The promoter region of the *CARTPT* gene contains binding sites for transcription factors such as AP2, SP1, and CREB, which selectively regulate basal and stimulus-induced (e.g., cAMP-mediated signaling pathway) expression (23, 34). *CARTPT* exhibits variable levels of expression in different tissues. Specifically, *CARTPT* is highly expressed in the arcuate nucleus of the hypothalamus. Lineage-specific transcription factors that bind enhancers are likely to contribute to the tissue-specific expression of *CARTPT*. However, the molecular mechanisms that restrict *CARTPT* expression and its silencing in tissues such as bone remain unknown.

Studies have implicated that CART peptides are involved in several physiological functions, including the regulation of reward/addiction (35, 36), food intake (37, 38), stress response (39), and anxiety/depression (40–42). Moreover, CART peptides have been reported to regulate bone remodeling (31, 43, 44). Elefteriou et al. showed that *Cartpt-*deficient mice (*Cartpt^–/–^*) with normal appetite and energy expenditure presented with osteoporosis due to increased bone resorption caused by upregulated *Rankl* expression in osteoblasts (31). Singh et al. further demonstrated that peripheral overexpression of *Cartpt* driven by *Col1a1-Cre* (herein *Col1a1-Cartpt*) could decrease osteoclastogenesis and rescue the low bone mass phenotype in *Cartpt^–/–^* mice (43). Together, these findings support a regulatory role of CART peptides in bone resorption.

Using preosteoblast-specific *Atrx* conditional knockout mice (*Osterix-Cre; Atrx^fl/y^*) with *Cre* expression postnatally, we observed increased trabecular bone mass and decreased osteoclast numbers when compared with *Atrx^fl/y^* control mice. In addition, we detected strong induction of *Cartpt* in the osteoblastic lineage cells. Given the regulatory role of CART peptides in bone resorption, we hypothesized that selective *Atrx* deletion in preosteoblasts might impair osteoclast differentiation by activating *Cartpt* expression. In this study, we investigated the biological role of ATRX in skeletal development at postnatal stages and uncovered an unexpected role for ATRX in the transcriptional silencing of *Cartpt* expression in osteoblastic lineage cells.

## Results

### Mice with specific Atrx deletion in preosteoblasts show decreased Atrx expression in bone, but display normal body weight and skeletal patterning.

To determine the functions of ATRX in skeletal development, we utilized *Osterix-Cre*, a preosteoblast-specific Cre recombinase, to generate conditional *Atrx*-knockout mice (*Osterix-Cre; Atrx^fl/y^* and *Osterix-Cre; Atrx^fl/fl^*, herein Atrx-cKO) at postnatal stages. Cre recombinase expression was prevented with doxycycline treatment in this Tet-off regulated recombinase line during embryonic development and preweaning. To examine the efficiency of *Atrx* deletion, we collected tibias from mice at 8 weeks of age, extracted RNA from the bones, and performed reverse transcription-PCR (RT-PCR). Atrx cKO tibias showed a reduction in *Atrx* expression when compared with *Atrx* floxed mice (*Atrx^fl/y^* and *Atrx^fl/fl^*, herein control) (Supplemental Figure 1A; supplemental material available online with this article; https://doi.org/10.1172/JCI163587DS1). Since the RNA was extracted from bulk bone tissues, the residual *Atrx* expression was attributed to the cells or tissues other than osteoblastic lineage cells. Additionally, we also observed a faint band with a smaller transcript size (254 bp) resulting from the recombination of *Atrx* exon 18 in Atrx-cKO (Supplemental Figure 1A). The observation of this smaller transcript was consistent with previous studies using this *Atrx* floxed mouse model (20, 22). Despite *Atrx* deletion, Atrx-cKO mice were born at the expected Mendelian ratios (45 control mice versus 47 Atrx-cKO mice, out of 23 litters). The observed ratios suggest no effect on fitness. Additionally, the growth curves of Atrx-cKO mice were comparable to those of control mice (Supplemental Figure 1B). Finally, radiograph showed normal skeletal patterning in both control and Atrx-cKO mice (Supplemental Figure 1C).

### Atrx deletion in preosteoblasts causes increased trabecular bone mass.

**Figure 1:**
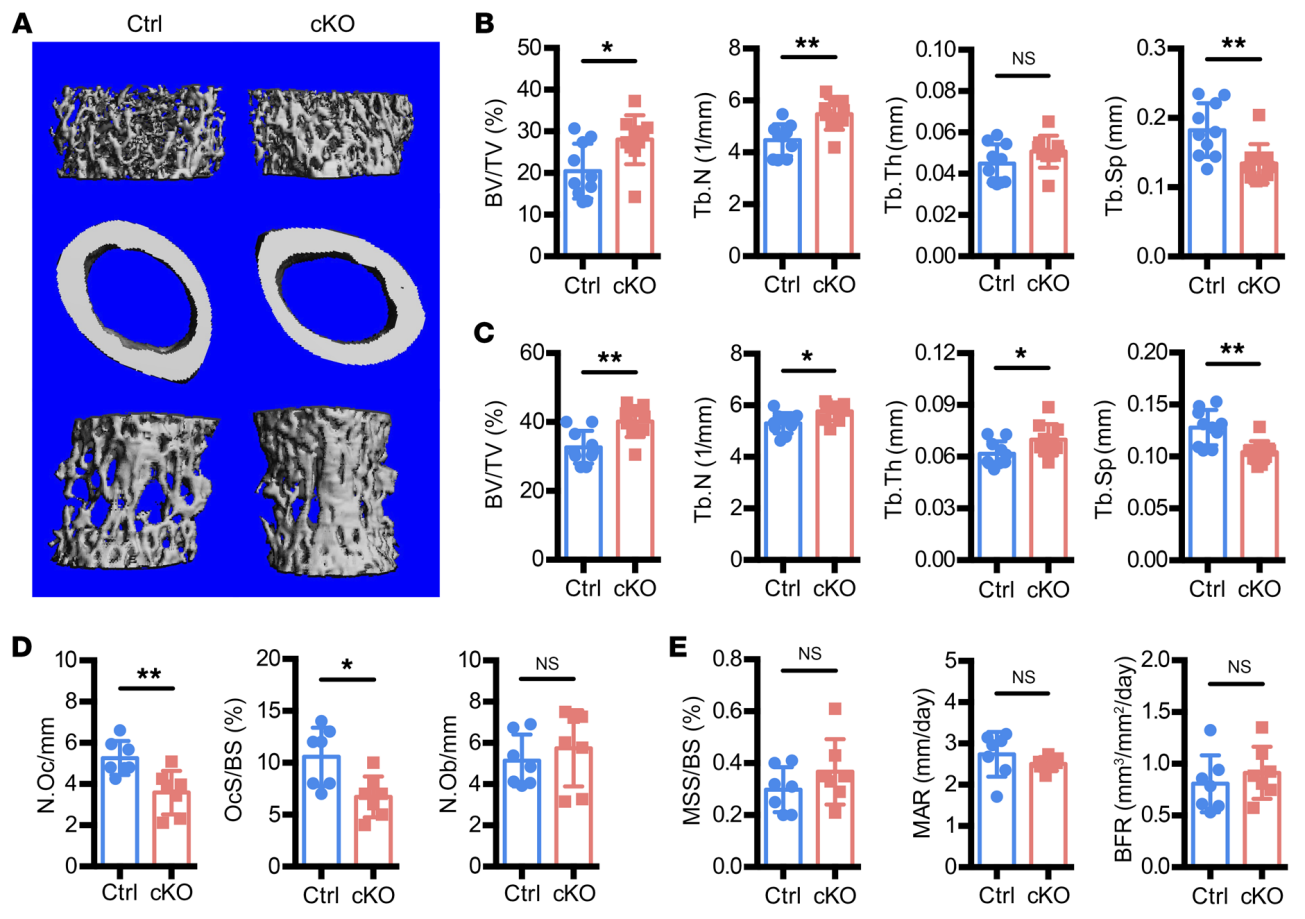
Deletion of *Atrx* in preosteoblasts causes increased trabecular bone mass. (**A**) Representative micro-CT images of control (Ctrl) and Atrx-cKO (cKO) mice at 8 weeks old. Top, trabecular bone at the distal femurs; middle, cortical bone at the midshaft femurs; bottom, trabecular bone at the fourth lumbar vertebrae. (**B** and **C**) Parameters of trabecular (BV/TV, Tb.N, Tb.Th, Tb.Sp) and cortical microarchitecture (Ct.Th) were analyzed in (**B**) femurs and (**C**) vertebrae of 8-week-old male mice. (**D**) Histomorphometric analysis of femurs in control and Atrx-cKO mice. (**E**) Dynamic histomorphometry analysis by calcein/alizarin red double labeling. BFR/BS, bone formation rate in control and Atrx-cKO mice. *n* = 7 per group. Data are represented as means with ± SD. Student’s *t* test. *P < 0.05; **P < 0.01.

To quantitatively evaluate bone architecture, we performed micro-CT analysis on 8-week-old control and Atrx-cKO male mice (Figure 1A). In Atrx-cKO, both femurs (Figure 1, A and B) and vertebrae (Figure 1, A and C) exhibited increased trabecular bone volume fraction (BV/TV) and trabecular number (Tb.N) and decreased trabecular spacing (Tb.Sp) (Figure 1B) when compared with control mice. In contrast, trabecular thickness (Tb.Th) in the long bones was not statistically different from control (Figure 1B). At the center of the femur midshaft, Atrx-cKO male mice showed no significant differences in cortical parameters including cortical thickness (Ct.Th), anterior-posterior diameter (diameter a.p.), and cross-sectional bone/marrow area (Supplemental Figure 2). These findings suggest that the effect of *Atrx* deletion in preosteoblasts is more robust in trabecular bone. Notably, Atrx-cKO male mice at 1.5 years of age also showed significantly increased Tb.N and decreased Tb.Sp as well as a trend for increased BV/TV (Supplemental Figure 3). In Atrx-cKO female mice, femurs exhibited increased trabecular bone mass while vertebrae showed an increased trend (Supplemental Figure 4). Cortical bone measurements of female mice did not show a difference between control and Atrx-cKO (Supplemental Figure 5), which is consistent with the male mice.

To exclude an effect from the expression of *Osterix-Cre* (45), we analyzed the skeletal phenotypes in *Osterix-Cre; Atrx^+/+^* (herein Osx) mice compared with *Atrx* floxed control. We did not see statistical differences in BV/TV, Tb.N, Tb.Th, Tb.Sp, and Ct.Th between Osx and control mice (Supplemental Figure 6). We also analyzed the expression of osteoblast markers (*Runx2*, *Col1a1*, and *Ocn*) in tibias and did not observe significant differences between Osx and control mice (Supplemental Figure 7, A–C). These results suggest that the Atrx-cKO bone phenotype was specific for *Atrx* deletion and not transgenic *Osterix-Cre* expression.

To investigate the cellular mechanism of increased trabecular bone in Atrx-cKO mice, we performed bone histomorphometric analysis using femurs. Atrx-cKO mice showed reductions in osteoclast number (N.Oc/mm) and osteoclast surface per bone surface (Oc.S/BS), but no change in osteoblast number per bone surface (N.Ob/mm) compared with control mice (Figure 1D). Dynamic histomorphometry analysis using double labeling showed comparable mineralizing surface per bone surface (MS/BS), mineral apposition rate (MAR), and bone formation rate (BFR) in both Atrx-cKO and control mice (Figure 1E). Together, these data suggest that decreased osteoclast numbers primarily contribute to the *Atrx* deletion–mediated increased trabecular bone mass.

### Atrx deletion in preosteoblasts decreases osteoclast differentiation that is associated with reduced Rankl/Opg expression ratio in BMSCs.

**Figure 2:**
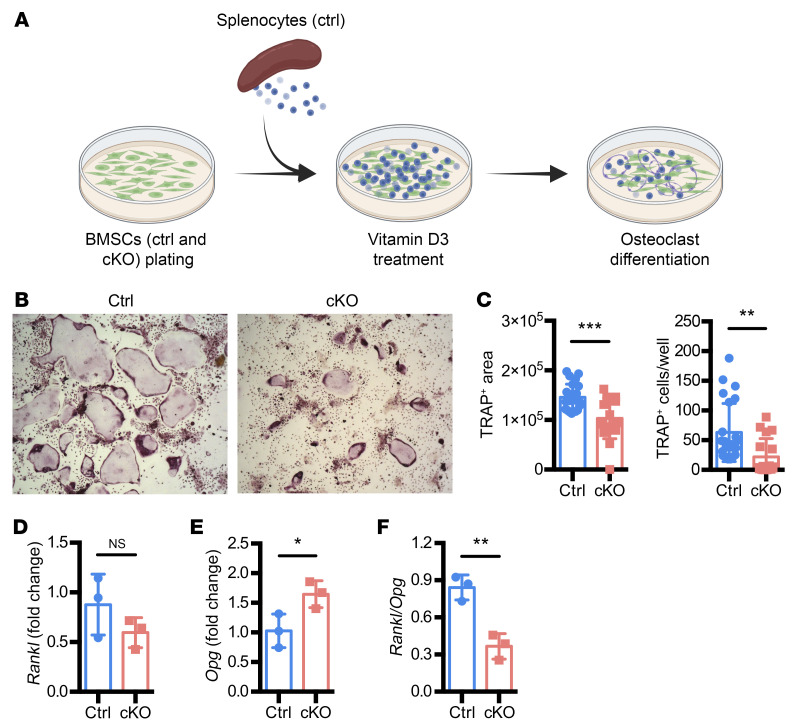
*Atrx* deletion in preosteoblast decreases osteoclast differentiation that is associated with reduced *Rankl/Opg* expression ratio in BMSCs. (**A**) Schematic illustration of in vitro osteoclast differentiation assay. Ctrl, cells isolated from 8-week-old Atrx^fl/y^ control mice; cKO, cells isolated from 8-week-old Atrx-cKO mice. (**B** and **C**) TRAP staining of multinucleated cells. Representative microscopic view of the cells after TRAP staining (**B**); quantification of TRAP-positive area and TRAP-positive cell number per well (**C**). Original magnification, ×5. *n* = 4 for *Atrx^fl/y^* control; *n* = 3 for Atrx-cKO. Six technical replicates were included in the quantification. (**D**–**F**) RT-qPCR results of *Rankl* (**D**), *Opg* (**E**), and *Rankl/Opg* ratio (**F**) in BMSCs from control and Atrx-cKO mice. *n* = 3 per group. Data are presented as means with ± SD. Student’s *t* test. *P < 0.05; **P < 0.01; ***P < 0.005.

Based on the results of the micro-CT and bone histomorphometry analyses, we hypothesized that the decreased osteoclast number observed in Atrx-cKO mice was due to decreased osteoclast differentiation. To test this hypothesis, we performed in vitro osteoclast differentiation assay by coculturing bone marrow stromal cells (BMSCs) isolated from 8-week-old control and Atrx-cKO male mice with Atrx WT splenocytes (Figure 2A). Atrx-cKO BMSCs were associated with a significant reduction in tartrate-resistant acid phosphatase–positive (TRAP-positive) area and TRAP-positive multinucleated cells per well (Figure 2, B and C), suggesting that *Atrx* deletion in BMSCs directly impairs osteoclast differentiation in the coculture assays. OPG and RANKL secreted by osteoblastic lineage cells have been known to regulate osteoclast differentiation (46). In the BMSCs isolated from Atrx-cKO mice, *Opg* expression was significantly increased, while *Rankl* was trending to decrease compared with the control (Figure 2, D and E). Together, this led to a reduced *Rankl/Opg* expression ratio in Atrx-cKO BMSCs (Figure 2F), suggesting an alteration in this primary biochemical determinant of bone resorption. Collectively, Atrx-cKO decreased osteoclast differentiation that was associated with reduced *Rankl/Opg* expression ratio.

### RNA-Seq analysis reveals Cartpt upregulation in Atrx-cKO mice.

**Figure 3:**
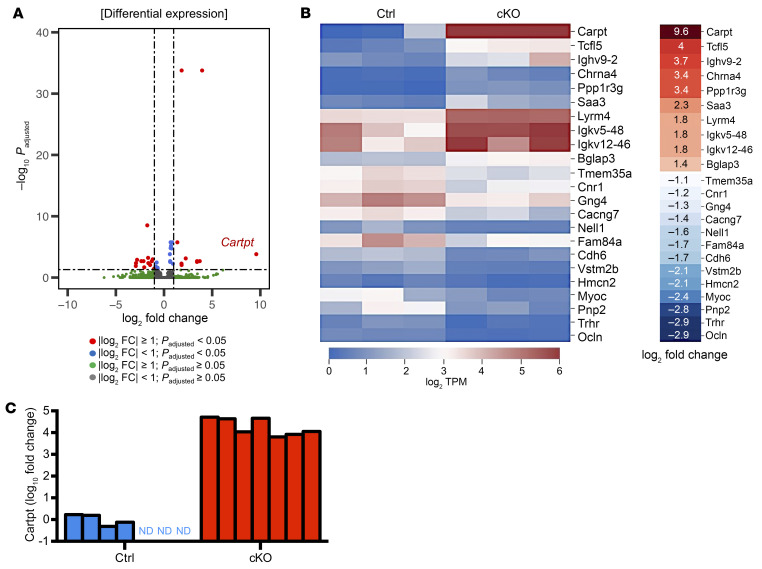
RNA-Seq analysis reveals *Cartpt* upregulation in Atrx-cKO mice. (**A**) Volcano plot of all DEGs identified from the DEG analysis with the thresholds of log_2_-fold change (FC) = 1 and *P_adjust_* value < 0.05. *Cartpt* showed high levels of expression with log_2_-FC = 9.6 and *P_adjust_* value < 0.0005. *n* = 3 per group. (**B**) Heatmap of all upregulated and downregulated DEGs color coded by log_2_ TPM and ranked by the log_2_ FC between the Atrx-cKO and control group. *n* = 3 per group. (**C**) RT-qPCR confirmation of RNA-Seq results for *Cartpt*. *n* = 7 per group. ND, not detected.

To understand the underlying mechanisms that impaired osteoclast differentiation, we performed RNA-Seq on the tibias harvested from control and Atrx-cKO mice at 8 weeks of age. By conducting analysis of differentially expressed genes (DEG), we identified 10 upregulated and 13 downregulated genes with the thresholds of log_2_-fold change = 1 and *P_adjust_* value < 0.05 (Figure 3, A and B). Among 23 DEGs, we found a high level of *Cartpt* expression in Atrx-cKO, with an average log_2_-fold change = 9.6. To validate the upregulation of *Cartpt* in Atrx-cKO, we performed quantitative reverse transcription–PCR (RT-qPCR) experiments on tibias from 7 control and 7 Atrx-cKO mice (Figure 3C). In the control group, *Cartpt* was not detectable in tibias from 3 out of 7 mice. In contrast, in the Atrx-cKO group, all samples showed more than 1,000-fold of *Cartpt* expression when compared with the control group. Gene ontology (GO) analysis of the upregulated gene sets in the Atrx cKO group further revealed significant enrichment in negative regulation of bone resorption (GO 0045779) (Supplemental Figure 8A). This result was consistent with the decreased Oc.S/BS phenotype in Atrx-cKO mice and the decreased *Rankl/Opg* expression ratio (Figure 1D and Figure 2F).

On the other hand, the analyses of the downregulated gene sets showed significant enrichment in negative regulation of osteoblast differentiation (GO 0033689) (Supplemental Figure 8B). We further measured the levels of *Runx2*, *Col1a1*, and *Ocn* expression in tibias and discovered increasing trends in all 3 genes in Atrx-cKO when compared with control mice (Supplemental Figure 9, A–C). However, osteoblast differentiation assays showed no difference in mineralization between control and the Atrx-cKO group (Supplemental Figure 9, D and E).

### Cartpt is highly expressed in the osteoblastic lineage cells of Atrx-cKO mice.

**Figure 4:**
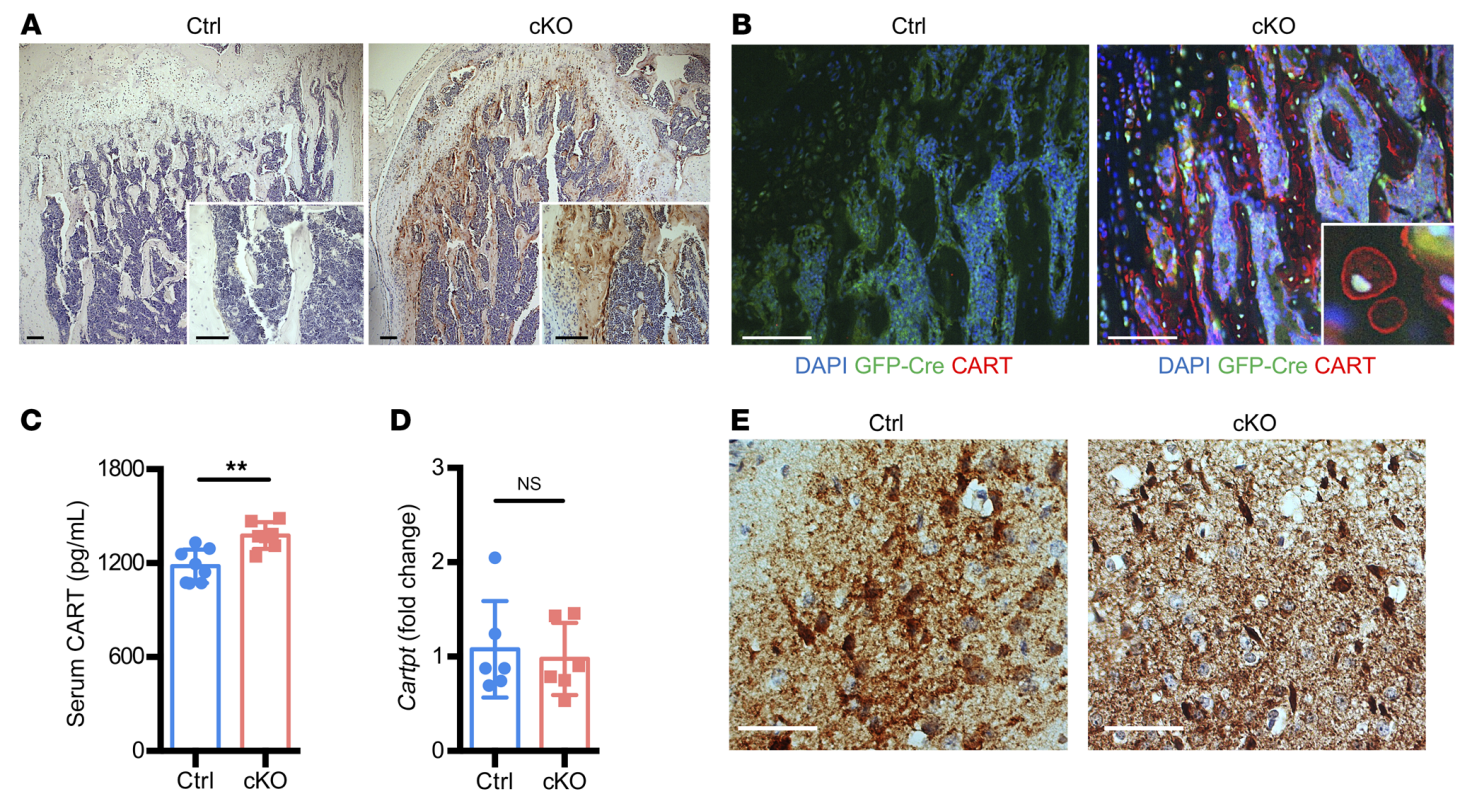
*Cartpt* is highly expressed in the osteoblastic lineage cells of Atrx-cKO mice. (**A** and **B**) (**A**) Images of CART peptides IHC staining in femurs at 8 weeks of age. High-magnification images are shown in white boxes on the bottom right. Scale bars: 100 μm. (**B**) IF stains for GFP-Cre fusion protein (green) and CART peptides (red) at 8 weeks of age in the control and Atrx-cKO. High-magnification image of CART-positive cells in Atrx-cKO is shown in white boxes on the bottom right. Scale bars: 50 μm. CART, CART peptides. (**C**) ELISA of serum CART peptides at 8 weeks of age. *n* = 8 for control; *n* = 7 for Atrx-cKO. (**D**) RT-qPCR results of *Cartpt* expression in hypothalamus. *n* = 6 per group. (**E**) CART peptides IHC staining in hypothalamus at 8 weeks of age. Scale bars: 100 μm. Data are represented as means with ± SD. Student’s *t* test. **P < 0.01. Representative images of *n* = 3 per group are shown.

As a neuropeptide, *CARTPT* is primarily expressed in hypothalamus (26). However, from previous studies and the Human Protein Atlas (www.proteinatlas.org) as well as our transcriptomic studies (Figure 3C), normal bone tissues do not express *CARTPT* (31–33). To examine the CART peptide levels in bone tissues, we performed CART peptides IHC staining on the femurs collected from both control and Atrx-cKO mice. Consistent with the observations in the RNA-Seq and RT-qPCR analyses shown in Figure 3, IHC staining revealed a strong staining of CART peptides in Atrx-cKO mice, whereas the signal was undetectable in control mice (Figure 4A). Notably, the CART peptide staining patterns were distributed in the ossification zone at trabeculae. Examining the images at higher magnification revealed high levels of CART peptides in lining cuboid, osteoblastic lineage cells residing on the surface of the bone (Figure 4A). To verify the CART peptide–expressing cell population, we examined the expression of GFP-Cre and CART peptides by immunofluorescence (IF) staining. The GFP-Cre fusion protein is under the control of the regulatory promoter of *Osterix* (47). Thus, the expression of the *GFP:Cre* transgene is restricted to the *Osterix*-expressing osteoblastic lineage and can be detected by anti-GFP staining. A large number of GFP-positive cells in Atrx-cKO mice were present at prehypertrophic/hypertrophic zones within the growth plate and ossification centers (Figure 4B), whereas no GFP signal was detectable in control mice (Figure 4B). The observations were consistent with the previous reports of endogenous *Osterix* expression in osteoblastic lineage cells (48). The signal of CART peptides colocalized with the GFP expression in the hypertrophic zones and bone trabeculae and overlapped *Osterix-Cre*–expressing cells (Figure 4B), supporting a cell-autonomous production of CART peptides. Notably, the CART peptide signal was prominently observed in the cell membrane of GFP-positive osteoblast-lineage cells (Figure 4B). Together, these results support the osteoblast-lineage cell origins of these CART peptide–expressing cells, suggesting a cell-specific effect of ATRX on *Cartpt* expression.

However, previous studies reported that local *Cartpt* overexpression in bone could lead to its secretion and systemic circulation (43). To compare the levels of circulating CART peptides, we performed ELISA to measure serum CART peptides in control and Atrx-cKO mice. The results of ELISA showed significantly increased serum CART peptides in Atrx-cKO mice at 8 weeks of age (Figure 4C). We also observed higher levels of serum CART peptides in Atrx-cKO when compared with control mice at 1.5 years of age (Supplemental Figure 10). These results suggest that *Atrx* loss–mediated *Cartpt* induction in osteoblasts contributes to the increase of CART peptides in circulation and this effect was sustained.

Since we detected increased circulating CART peptides in Atrx-cKO mice, we asked whether the increased CART peptides in blood might circulate to the brain and act in a regulatory loop to modulate *Cartpt* expression in the hypothalamus. RT-qPCR experiments showed that there were no changes in the *Cartpt* expression in the hypothalamus between control and Atrx-cKO mice (Figure 4D). CART peptide IHC staining at the hypothalamus further supported similar levels of CART peptides in both control and Atrx-cKO mice (Figure 4E).

### ATRX and RUNX2 bind to the regulatory regions of Cartpt.

**Figure 5:**
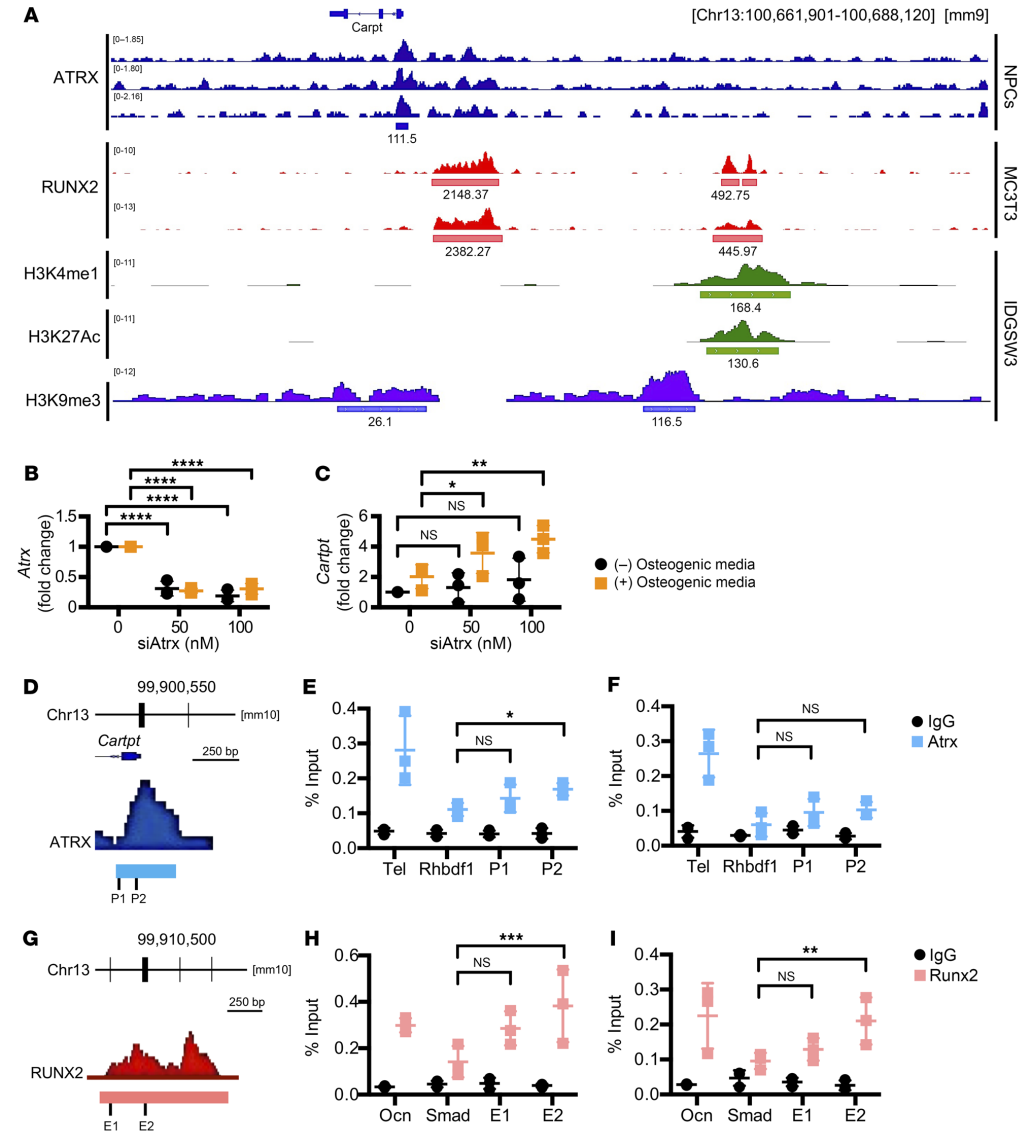
ATRX and RUNX2 bind to the regulatory regions of *Cartpt*. (**A**) Genome browser representations of published ATRX, RUNX2, H3K4me1, H3K27Ac, and H3K9me3 ChIP-Seqs near *Cartpt*. ATRX, ATRX ChIP-Seq in mouse NPCs. RUNX2, RUNX2 ChIP-Seq in differentiated MC3T3 cells. H3K4me1/H3K27Ac/H3K9me3, H3K4me1, H3K27Ac, and H3K9me3 ChIP-Seqs in IDGSW3 cells. Data represented as read density in reads normalized to 10^8^. Blue, pink, and green boxes indicate peak regions. The peak score under each color box was based on peak calling analysis. (**B** and **C**) RT-qPCR results of *Atrx* (**B**) and *Cartpt* (**C**) expression in MC3T3 cells transfected with 100 nM siControl (shown as 0 nM) or siAtrx (shown as 50 nM and 100 nM). Black, cells at the undifferentiated state; orange, cell differentiating for 3 days in osteogenic media. *n* = 3 per group. Data are presented as means with ± SD. Two-way ANOVA. *P < 0.05; **P < 0.01; ****P < 0.001. (**D**–**F**) ATRX ChIP-qPCR in MC3T3 cells. Primer design at the edge (P1) or peak (P2) of the ATRX binding near the *Cartpt* promoter region (**D**). ATRX ChIP-qPCR in MC3T3 cells at undifferentiated (**E**) and differentiated (**F**) (cell differentiating for 5 days) state. Tel, positive control of ATRX bindings at telomere; Rhbdf1, negative control of ATRX bindings at the *Rhbdf1* intron region. *n* = 3 per group. (**G**–**I**) RUNX2 ChIP-qPCR in MC3T3 cells. Primer design at the edge (E1) or peak (E2) of the RUNX2 binding 10 kbp upstream of the *Cartpt* promoter (**G**). RUNX2 ChIP-qPCR in MC3T3 cells at undifferentiated (**H**) and differentiated (**I**) (cell differentiating for 5 days) state. Ocn, positive control of RUNX2 bindings at the *Ocn* promoter; Smad, negative control of RUNX2 bindings at the *Smad4* intron region. *n* = 3 per group. Data are represented as means with ± SD. Two-way ANOVA. *P < 0.05; **P < 0.01; ***P < 0.005.

Since *Cartpt* is physiologically silenced in bone tissues, we asked how *Atrx* deletion in preosteoblasts was sufficient to induce high *Cartpt* expression in osteoblast-lineage cells. Previous studies showed that ATRX could act as a transcription regulator by recruiting transcriptional coactivators/repressors and by modulating local chromatin structures (2, 3, 12, 49). Therefore, we hypothesized that ATRX binding to *Cartpt* regulatory elements could directly suppress *Cartpt* expression. To determine whether ATRX interacts with potential gene regulatory elements near *Cartpt*, we examined the ATRX occupancy within ±5 kbp of the transcription starting site (TSS) of *Cartpt* by analyzing the published dataset of ATRX chromatin immunoprecipitation sequencing (ChIP-seq) (NCBI’s Gene Expression Omnibus [GEO] database GSE100462) (50). Interestingly, we discovered an ATRX-binding peak near the TSS of *Cartpt* (Figure 5A). Furthermore, the dataset from the Encyclopedia of DNA Element (ENCODE) Data Analysis Center characterized this ATRX-binding site as a promoter-like region (EM10E0570099) (51). The analysis of the published H3K9me3 ChIP-Seq datasets (GEO GSE54782) (52) in IDGSW3 cells (mouse osteoblast cell line) showed enrichment in the *Cartpt* promoter region, indicating less active and condensed chromatin (Figure 5A). Both ATRX and H3K9me3 ChIP-Seq analyses suggest that ATRX could act as a transcription regulator by binding to the *Cartpt* promoter and repressing the transcription of *Cartpt*.

Given the in vitro observations suggest a cell-autonomous effect of ATRX as a suppressor on *Cartpt* expression (Figure 4B), we probed for potential transcriptional activators, such as RUNX2, that may mediate *Cartpt* induction in osteoblastic lineage cells. By analyzing publicly available RUNX2 ChIP-Seq datasets (GSM1305863) (53) from differentiated MC3T3 cells (mouse preosteoblast cell line) and examining RUNX2 binding near *Cartpt* (±12 kbp of TSS), we discovered 2 genomic loci with RUNX2 binding at 2.5 kbp (proximal site) and 10 kbp (distal site) upstream of the *Cartpt* promoter (Figure 5A). Transcription factor binding motif (TFBM) analysis further identified the consensus motif (TGNGGTT) of the RUNX family at the distal site (54). Furthermore, the analyses of the published active enhancer histone marks H3K4me1 and H3K27Ac ChIP-Seq datasets (GEO GSE54782) (52) in differentiated IDGSW3 cells showed strong enrichment at these distal binding sites (Figure 5A). Both TFBM and H3K4me1/H3K27Ac ChIP-Seq analyses supported that the RUNX2 binding at these distal sites could be a potential enhancer poised to activate *Cartpt* transcription that is actively repressed by H3K9me3 enrichment and ATRX recruitment during normal skeletal development.

To further test this hypothesis, we performed ATRX/RUNX2 ChIP-qPCRs in MC3T3 cells to validate the protein-DNA interactions. MC3T3 exhibits low levels of *Cartpt* in the undifferentiated state (Supplemental Table 1), which acts as an ideal cell model for examining the effects of *Atrx* silencing on *Cartpt* induction. We first examined whether siRNA-mediated *Atrx* knockdown induced *Cartpt* expression in MC3T3 (Figure 5, B and C). By knocking down *Atrx*, we observed a dose-dependent increase on *Cartpt* expression when differentiating in osteogenic media (Figure 5C). The results confirmed that we were able to recapitulate the in vivo induction of *Cartpt* expression by transient *Atrx* knockdown in vitro. By conducting ATRX ChIP-qPCR (Figure 5, D–F), we identified a significant enrichment of ATRX signal at the “peak” (P2) region within the *Cartpt* promoter in undifferentiated (Figure 5E) but not in differentiated states (Figure 5F) compared with the negative control (*Rhbdf1* intron). The weaker level of ATRX signal was also measured at the “edge” (P1) in both undifferentiated and differentiated states, but the enrichment was not significant. In the RUNX2 ChIP-qPCR (Figure 5, G–I), we measured a strong enrichment of RUNX2 binding at the putative *Cartpt* enhancer (E2), similar to the level observed in the positive control at the *Ocn* promoter, in both undifferentiated (Figure 5H) and differentiated (Figure 5I) states. Differentiation into osteoblasts did not alter the magnitude of RUNX2 binding at the *Cartpt* enhancer in our MC3T3 model. This suggests that RUNX2 as a transcription factor poised for transactivation that requires active relief of repression by removal of ATRX. Collectively, the ChIP-qPCR data corroborates the gene regulatory mechanisms mediated by the ATRX and RUNX2 binding at the *Cartpt* promoter and the enhancer, respectively.

### Cartpt loss increases Rankl/Opg expression ratio in Atrx-cKO BMSCs.

**Figure 6:**
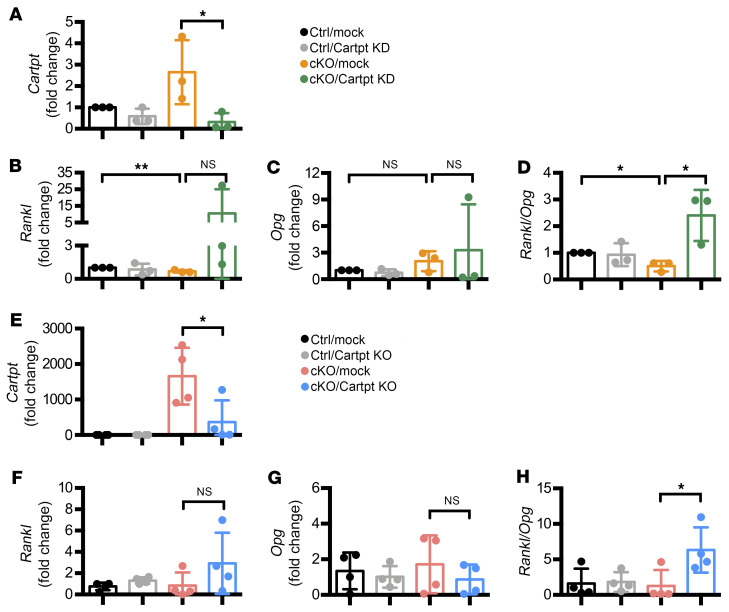
*Cartpt* loss increases *Rankl/Opg* ratio in Atrx-cKO BMSCs. (**A**–**D**) RT-qPCR results of *Cartpt* (**A**), *Rankl* (**B**), *Opg* (**C**), and *Rankl/Opg* ratio (**D**) in BMSCs isolated from control (Ctrl) and Atrx-cKO (cKO) mice. The cells were transfected with 10 nM siControl (mock) or 10 nM siCartpt (Cartpt KD). After overnight incubation, the cells were cultured in the osteogenic media for 3 days. *n* = 3 per group. (**E**–**H**) RT-qPCR results of *Cartpt* (**E**), *Rankl* (**F**), *Opg* (**G**), and *Rankl/Opg* (**H**) expression ratio in BMSCs isolated from Ctrl and cKO mice. The cells were transfected with 1 μg of the pegRNA plasmid (mock or Cartpt KO) and 0.5 μg of pCMV-PE2-GFP. The transfected cells were treated with osteogenic media for 3 days. *n* = 4 per group. Data are represented as means with ± SD. One-way ANOVA. *P < 0.05; **P < 0.01.

We observed a decrease in *Rankl*/*Opg* expression ratio (Figure 2F) associated with induced *Cartpt* expression in the Atrx-cKO mice (Figure 3C). This led us to examine whether CART peptides were negative regulators for bone resorption through the regulation of *Rankl* and *Opg* expression. To examine the regulatory role of CART peptides in bone resorption, we knocked down *Cartpt* in the Atrx-cKO BMSCs to test the effects on the *Rankl*/*Opg* expression ratio. The *Cartpt* siRNA transfected BMSCs were subsequently differentiated in osteogenic media for 3 days. RT-qPCR experiments confirmed a successful *Cartpt* knockdown in both control and Atrx-cKO BMSCs (Figure 6A). In Atrx-cKO BMSCs transfected with control siRNA (siControl), *Rankl* expression was significantly decreased, while *Opg* was trending to increase compared with mock control BMSCs (Figure 6, B and C). This led to a reduced *Rankl/Opg* expression ratio in mock Atrx-cKO BMSCs (Figure 6D). Upon *Cartpt* knockdown, both *Rankl* and *Opg* expression showed increasing trends in Atrx-cKO BMSCs (Figure 6, B and C). The combined effects on *Rankl/Opg* expression ratios were significantly upregulated after knocking down *Cartpt* (Figure 6D). Control BMSCs did not show any significant changes in *Rankl*, *Opg*, and *Rankl/Opg* ratio (Figure 6, B–D). To further confirm these findings, we generated *Cartpt*-knockout BMSCs by CRISPR-prime editing in Atrx-cKO BMSCs. With high efficiency in *Cartpt* deletion (Figure 6E), *Rankl* showed an increasing trend while *Opg* expression revealed a decreasing trend in Atrx-cKO BMSCs (Figure 6, F and G). Together, these led to an increased *Rankl/Opg* expression ratio in Atrx-cKO BMSCs (Figure 6H), similar to the observation in siRNA-mediated *Cartpt* knockdown conditions (Figure 6D). These results suggest that *Cartpt* loss in Atrx-cKO BMSCs results in an increased *Rankl/Opg* expression ratio. Finally, we generated founder mice of *Cartpt* knockout in the background of Atrx-cKO by CRISPR/Cas9 to further study the epistatic interaction of ATRX and CARTPT on bone mass. Based on micro-CT analysis on these founder mice, *Cartpt* deletion showed a trend toward to decreased trabecular bone volume in femurs in a dosage-dependent manner (biallelic showed a greater reduction of bone mass compared with monoallelic *Cartpt* deletion) (Supplemental Figure 11). Collectively, our study of a *Osterix*-*Cre*–driven *Atrx* deletion mouse model demonstrates that ATRX actively suppresses *Cartpt* expression in osteoblasts during physiological bone development by regulating bone resorption via modulating the *Rankl*/*Opg* expression ratio.

## Discussion

In this study, we show that postnatal deletion of *Atrx* in preosteoblasts increases trabecular bone by impairing osteoclast differentiation which is accompanied by reduced *Rankl/Opg* expression ratio. We further show that specific *Atrx* deletion induces high levels of *Cartpt* expression in osteoblasts. However, *Atrx* deletion in preosteoblasts does not alter *Cartpt* expression in hypothalamus. Importantly, we confirm that these *Cartpt*-expressing cells overlap with GFP-positive osteolineage cells harboring *Atrx* deletion, indicating that *Atrx* deletion specifically leads to the *Cartpt* induction in a cell-specific fashion, although CART peptides could also work in paracrine and/or endocrine fashion. Mechanistically, deleting *Atrx* in osteoblasts displaces the binding of ATRX in the *Cartpt* promoter region. This ATRX binding along with H3K9me3 modification in silico at the *Cartpt* promoter would be consistent with its inhibition of aberrant expression of *Cartpt*. As a putative distal enhancer is occupied by RUNX2, *Cartpt* would require active repression given it is poised for expression in osteoblastic cells. Lastly, *Cartpt* loss by siRNA and CRISPR-prime editing in *Atrx* cKO BMSCs increases the *Rankl/Opg* expression ratio, confirming the role of CART peptides in regulating bone resorption. Together, we demonstrated a potent regulation of bone resorption mediated by ATRX and its transcriptional regulation on *Cartpt*.

Based on bone micro-CT analysis, we discovered that *Atrx* loss increases trabecular bone but not cortical bone in both male and female mice (Figure 1B, Supplemental Figure 2, Supplemental Figure 4, and Supplemental Figure 5). This discrepancy could be attributed to a more active bone remodeling in trabecular bone (55), where osteoblasts and osteoclasts actively differentiate. During early osteoblast differentiation, RUNX2 governs the differentiation of preosteoblasts into mature osteoblasts. In osteoblastic lineage cells, upon the depletion of *Atrx* at the promoter region of *Cartpt*, the binding of RUNX2 at the distal enhancer of *Cartpt* could drive its expression and result in the accumulation of CART peptides in the trabecular bone (Figure 4A). Ultimately, the accumulation of CART peptides in bone cells would inhibit osteoclastogenesis in the trabecular bone. Together, the differential expression of *Cartpt* and its role in suppressing bone resorption led to varied phenotypes in 2 different bone compartments.

The gene ontology analyses of the downregulated gene sets show significant enrichment in negative regulation of osteoblast differentiation (Supplemental Figure 8B), indicating potential impact of Atrx conditional deletion on osteoblast. RT-qPCR experiments further showed increasing trends in *Runx2*, *Col1a1*, and *Ocn* expression in Atrx-cKO mice (Supplemental Figure 9, A–C). Although the transcriptomic characteristics favor osteoblast differentiation in the context of *Atrx* deletion, the osteoblast mineralization assays did not show effects on mineralization (Supplemental Figure 9, D and E). Together, these results may suggest a more complex but subtle effect of ATRX on osteoblast differentiation.

Several transcription factors, including AP2, SP1, and CREB, have been found to bind to the *Cartpt* promoter and regulate *Cartpt* expression in neuronal cells (23, 34). However, it is unclear how *Cartpt* is regulated and silenced in bone cells. In our study, we discovered unique ATRX binding to the *Cartpt* promoter (Figure 5), which functions as a transcriptional repressor for *Cartpt* expression. The ATRX cobinding with DAXX at the CMV promoter has been reported by Newhart et al. (56). They showed that *ATRX* deletion robustly induced CMV-mediated transgene expression, suggesting a transcriptional repressive role of ATRX in gene regulation. Consistent with their finding, we observed a drastic increase of *Cartpt* expression in the bone of Atrx-cKO models from normally undetectable levels. The precise molecular mechanism by which ATRX suppresses *Cartpt* expression remains unclear. One possible mechanism is that ATRX recruits additional bone-specific repressors to the regulatory regions of *Cartpt*, preventing aberrant *Cartpt* expression in the bone. As ATRX binding to *cis*-regulatory elements is linked to alterations in chromatin status (57), ATRX could alternatively form a complex with other epigenetics regulators to remodel local chromatin status near *Cartpt* during osteoblast differentiation. In our study, we show the enrichment of ATRX in the promoter region of *Cartpt* (Figure 5, E and F). With ATRX occupancy and H3K9me3 modification observed in published ChIP-Seq datasets (Figure 5A), *Cartpt* remains silenced in the osteoblastic cells in a physiological context. Since ATRX has also been reported to modulate higher-order chromatin interactions with cohesin and CTCF (CCCTC-binding factor) (49, 58, 59), it is also possible that loss of ATRX disrupts chromatin looping and results in changes in expression of genes (such as *Cartpt*) in *cis* or *trans*. Further studies into the molecular mechanism of ATRX, as a transcriptional repressor, in modulating *Cartpt* expression will provide additional mechanistic insights into cell-type/tissue-specific and universal gene regulation of ATRX.

CART peptides have been reported to play an important role in bone resorption. *Cartpt^–/–^* mice display a low bone mass phenotype resulting from increased *Rankl* expression and osteoclast number (31). Additionally, peripheral overexpression of *Cartpt* driven by the *Col1a1* promoter (Col1a1-Cartpt) rescues the low bone mass phenotype of *Cartpt^–/–^* mice (43). Consistent with these findings, we discovered decreased osteoclast differentiation and reduced *Rankl/Opg* expression ratio resulted in increased bone mass in the context of *Atrx* deletion–mediated *Cartpt* activation (Figure 1 and Figure 2). Moreover, *Cartpt* loss rescued the low *Rankl/Opg* expression ratio in Atrx-cKO BMSCs (Figure 6). Consistent with this in vitro study, *Cartpt* deletion in Atrx-cKO mice showed a trend toward decreased trabecular bone volume in femurs in a dosage-dependent manner from the founders (Supplemental Figure 11). Collectively, our data corroborate the role of CART peptides in regulation of osteoclast differentiation, and, for the first time, to our knowledge, we demonstrate ATRX as an upstream regulator of *Cartpt* in osteoblasts.

Our current discovery of ATRX-CART peptide function in bone remodeling raises interesting questions for future directions. As 1.5-year-old Atrx -cKO male mice showed high bone mass (Supplemental Figure 3), would the female mice also be protected against bone loss with aging? This may point to a regulatory mechanism that is important in designing treatment of osteoporosis. Extensive future studies on osteoporosis using ovariectomy (OVX) models are required to study the underlying mechanisms. In addition, what are the potential cell populations responding to CART peptides that further modulate the expression of *Rankl/Opg*? Several studies have suggested G-protein–coupled receptor coupled to inhibitory G protein (Gi/o) as the receptor of CART peptides (60, 61). However, the actual receptors for CART peptides are still unknown. Other effects of ATRX on regulating the potential downstream mediators of CART peptides, such as at the receptor level, are also unknown. Future studies are warranted to identify the actual receptors of CART peptides for better determining the downstream pathway of CART peptides and further developing antagonists and agonists for future studies and the therapeutic purpose of regulating the bone remodeling.

## Methods

### Sex as a biological variable.

Our study examined both male and female mice, and similar findings were reported for both sexes. As the individuals affected by ATR-X syndrome are almost exclusively males, our study focuses more on male mice.

### Mice.

*Atrx*-floxed mice (62) were provided by Jason Huse at University of Texas MD Anderson Cancer Center (Houston, Texas, USA). To generate osteoblast progenitor-specific *Atrx*-knockout mice, *Osterix-Cre* transgenic (B6.Cg-Tg[Sp7-tTA,tetO-EGFP/cre]1Amc/J; Jackson Laboratory, 0063631) mice (47) were crossed with *Atrx*-floxed mice. The mice were maintained under doxycycline treatment to suppress Cre-mediated recombination from conception until 3 weeks of age. Biallelic or monoallelic *Cartpt*-knockout mice were generated by CRISPR/Cas9 gene targeting (guide sequence: CTCGTGGGACGCATCATCCA) in the background of *Atrx*-cKO. PCR genotyping was performed from ear-derived genomic DNA. For genotyping *Atrx*, the band size of WT and floxed are 1.0 kb and 1.5 kb, respectively (50); for *Cre*, the size is 198 bp (Jackson Laboratory). Eight-week-old tibias were collected for RT-PCR for Atrx expression analysis in bone (20). Primer sequences are listed in Supplemental Table 2.

### Cell culture.

The mouse preosteoblast cell line MC3T3 (MC3T3-E1) was purchased from ATCC. The cells were maintained in α-MEM supplemented with 10% fetal bovine serum, 1% l-glutamine, and 1% penicillin and streptomycin. To induce osteogenic differentiation, cells were cultured in osteogenic media: a complete α-MEM added to 500 μM ascorbic acid (Sigma-Aldrich) and 5 mM β-glycerophosphate (Sigma-Aldrich). Media were replaced every other day. The complete differentiation days are described in Results.

To generate BMSCs, bone marrow cells were isolated from tibia and femur of 8-week-old mice and cultured in complete α-MEM. Media was replaced every other day to remove nonadherent cells. After 1 week, the attached cells were characterized as BMSCs for subsequent osteoclast differentiation assays. For osteoclast differentiation, BMSCs were seeded in plates. On the next day, BMSCs were cocultured with splenocytes (the seeding ratio of BMSCs to splenocytes is 1:10) in complete α-MEM supplemented with 10 nM vitamin D3 (Enzo Life Science) for 4–5 days. For splenocyte isolation, a fresh whole spleen was dissected from 1 *Atrx^fl/y^* mouse and placed into a cell strainer. With the plunger end of a syringe, the spleen was mashed and pressed through the strainer. Splenocytes were washed and resuspended in PBS. After 5-minute centrifugation at 300*g*, splenocytes were resuspended in α-MEM. TRAP staining was performed using the Acid Phosphatase Leukocyte Kit (Sigma-Aldrich) according to the manufacturer’s instructions. Osteoclast differentiation was evaluated by quantifying the area and the number of TRAP-positive multinucleated cells by ImageJ software (NIH). For osteoblast differentiation, BMSCs were differentiated in osteogenic media as described above. After 21 days, the cells were fixed in 4% paraformaldehyde and subjected to alizarin red S staining (Sigma-Aldrich). The alizarin red S concentration was determined by absorbance measurement at 562 nm.

### siRNA-mediated knockdown.

MC3T3 or BMSCs were seeded in plates. The following day, cells were transfected with siControl (siGENOME Non-Targeting Control siRNA Pool 1; Dharmacon), siAtrx (siGENOME Mouse Atrx siRNA pool; Dharmacon), or siCartpt (siGENOME Mouse Cartpt siRNA pool; Dharmacon) using lipofectamine RNAiMAX (Thermo Fisher Scientific) according to the manufacturer’s instructions. The siRNA concentration is indicated in Results.

### Generation of Cartpt-knockout BMSCs by prime editing.

An in-frame stop codon TAG was designed to insert in the exon 1 of *Cartpt* by the prime editor. The spacer and extension templates of the prime editing guide RNA (pegRNA) were designed by PrimeDesign (https://github.com/pinellolab/PrimeDesign). The pegRNA was cloned into the pU6-pegRNA-GG-acceptor vector (Addgene, 132777) based on the protocol published by Anzalone et al. (63). To generate the *Cartpt*-knockout BMSCs, cells seeded in a 6-well plate were transfected with 1 μg of the pegRNA plasmid and 0.5 μg of pCMV-PE2-GFP (Addgene, 132776) using Xfect Transfection Reagent (Clontech Laboratories). The transfection mixture for each well was prepared in 1.5 μl Xfect Polymer and 100 μl Xfect Reaction Buffer according to the manufacturer’s instructions. Media was replaced 4 hours after transfection. After overnight incubation, the transfected cells were treated with osteogenic media. The cells were further grown for 72 hours and harvested for *Cartpt* knockout validation and *Rankl*/*Opg* expression analysis by RT-qPCR. The sequences of the pegRNA are listed in Supplemental Table 2.

### Gene expression analysis by RT-qPCR.

Total RNA was extracted with TRIzol (Thermo Fisher Scientific). iScript Reverse Transcription Supermix (Bio-Rad) was used for complementary DNA synthesis according to the manufacturer’s instructions. RNA expression was analyzed by qPCR with SYBR Green I reagent (Roche). *B2m* (β2 microglobulin) was used as a reference gene for normalization. Primer sequences are listed in Supplemental Table 2.

### Radiograph imaging, skeletal analysis, and bone histomorphometry.

X-ray radiography was performed with the Kubtec XPERT80 system. Eight-week-old spines and femurs were collected for quantification of trabecular and cortical bone parameters by micro-CT (Scanco Micro-CT40 System). After they were micro-CT scanned, undecalcified femurs were subsequently embedded in plastic for sectioning. Trichrome and TRAP staining were performed for visualization of osteoblasts and osteoclasts, respectively. Calcein/alizarin red double labeling was used for dynamic histomorphometry. Parameters of bone formation and resorption were analyzed by the BIOQUANT Osteo Image Analysis System.

### Histology and immunostaining.

Femurs were dissected and fixed with 10% neutral buffered formalin for 48 hours with gentle shaking. The samples were decalcified using Immunocal Decalcifier (Statlab) for 48 hours, followed by paraffin embedding and then sectioning. For hypothalamus, tissues were fixed with 4% paraformaldehyde for 24 hours at 4°C and then subjected to paraffin embedding. For CART peptide IHC staining, after deparaffinization and rehydration, sections were treated with 0.05% trypsin followed by 3% hydrogen peroxide treatment. After further blocking with 5% normal goat serum, sections were incubated with rabbit anti-CART peptides (Phoenix Pharmaceuticals, H-003-62) overnight at 4°C according to the manufacturer’s instructions. The following day, anti-rabbit secondary antibody (Vectastain ABC system, Vector Laboratories) was applied, and signal was developed using 0.1% 3, 39-diamino-benzidine. For IF staining of GFP-Cre fusion protein and CART peptides, the sections were treated with 0.05% trypsin and then blocked with 3% normal serum for 1 hour. Slides were then incubated with primary antibodies (chicken anti-GFP antibody, abcam, ab13970; rabbit anti-CART peptides, Phoenix Pharmaceuticals, H-003-62) overnight at 4°C. The following day, sections were incubated with secondary antibody (goat anti-chicken secondary antibody conjugated to Alexa Flour 488, Invitrogen, A11039; donkey anti-rabbit secondary antibody conjugated to Alexa Flour 594, Invitrogen, A21207) for 1 hour. The slides were mounted with ProLong Gold Antifade Reagent with DAPI (Invitrogen). All immunostaining images were taken using a Zeiss Axioskop40 microscope (Axiovision Software).

### CART peptides level by ELISA.

Blood samples were collected from 8-week-old and 1.5-year-old mice by orbital-sinus bleeding under anesthesia before euthanasia. Serum was separated by centrifuge in the tube with serum separator (BD Microtainer) and stored at –80°C. The assay was performed using the RayBio CART Enzyme Immunoassay Kit (RayBiotech) according to the manufacturer’s instructions.

### RNA-Seq and analysis.

Snap-frozen tibias and hypothalamus were pulverized using Tissuelyser II (QIAGEN). Total RNA was extracted using cold chloroform and precipitated in cold ethanol. RNA samples were further purified with the Direct-zol RNA Kit (Zymo Research). RNA quality and quantity were measured by Bioanalyzer (Agilent Technologies). Library preparation, sequencing with Illumina HiSeq (150-bp paired-end reads), and differential gene expression analysis were performed by GENEWIZ. Gene expression data were normalized per million transcripts (transcripts per million [TPM]). Gene ontology (biological process) analysis was conducted using Enrichr (64). Twenty-three DEGs are listed in Supplemental Table 3.

### ChIP, ChIP-qPCR and ChIP-seq analysis.

MC3T3 cells were fixed with 1% formaldehyde (final concentration) in α-MEM. Fragmented chromatin was prepared with the SimpleChIP Enzymatic Chromatin IP Kit (Cell Signaling Technology) according to the manufacturer’s instructions. ChIPs were performed with rabbit anti-ATRX (abcam, ab97508), rabbit anti-RUNX2 (abcam, ab236639), and normal rabbit IgG (negative control, Cell Signaling Technologies, 2729) for overnight incubation at 4°C. Immunoprecipitated DNA was quantified by qPCR using SYBR Green I Reagent (Roche). Primer sequences are listed in Supplemental Table 2.

Public ATRX, RUNX2, H3K4me1, H3K27Ac, and H3K9me3 ChIP-Seq datasets and peak calling score were downloaded from the NCBI database: ATRX ChIP-seq in mouse neuroepithelial progenitors (NPCs) (GEO GSE100462) (50); RUNX2 ChIP-seq in MC3T3 cells (GEO GSM1305864 and GSM1305865) (53); H3K4me1, H3K27Ac, and H3K9me3ChIP-seqs in IDGSW3 cells (GEO GSM1323923, GSM1323926, and GSE54782) (52). The Integrative Genomics Viewer (IGV) was used to visualize the enrichment of ATRX, RUNX2, H3K4me1, and H3K27Ac near *Cartpt*. The TSS and promoter of *Cartpt* were defined based on the characterization from the ENCODE project (51). Data were represented as read density in reads normalized to 10^8^.

### Statistics.

All data are presented as means with ± SD. The statistical methods and significance criteria are listed in Results. Analyses involving 2 groups with replicates were subjected to unpaired, 2-tailed Student’s *t* test. Comparisons among multiple groups were performed by 2-way ANOVA test. GraphPad Prism, version 6.0, was used for all statistical analysis.

### Data availability.

RNA-Seq data files were deposited in the NCBI’s Gene Expression Omnibus (GEO GSE273097). The full unedited gel image for Supplemental Figure 1 is included in the Supplemental Material. Representative images of secondary antibody controls for IHC and IF staining are shown in Supplemental Figures 12 and 14, respectively. Representative images of ATRX IHC staining in trabecular and cortical bone compartments in *Atrx*^fl/y^ mice are shown in Supplemental Figure 13. Primer sequences are listed in Supplemental Table 2. A list of 23 DEGs are included in Supplemental Table 3. Values for all data points in graphs are reported in the Supporting Data Values file.

### Study approval.

All animal care and experimental procedures were approved by the Institutional Animal Care and Use Committee (IACUC) of Baylor College of Medicine, and experiments were performed in compliance with the Baylor College of Medicine Animal Protocol AN5136.

## Author contributions

YTC, YB, and BHL conceived and designed the study. YTC, MMJ, CL, MA, CFM, BI, UP, GH, ZJ, DGL, LL, BD, YCE, OER, RJG, and JDH conducted the experiments. YTC analyzed data. YTC wrote the manuscript. YTC, YB, and BHL reviewed and edited the manuscript with input from all authors. BHL acquired funding. YB and BHL supervised the project.

## Supplementary Material

Supplemental data

Unedited blot and gel images

Supporting data values

## Version 1 01/02/2025

Electronic publication
