# Supplementary material for: ATRX silences Cartpt expression in osteoblastic cells during skeletal development
Source: J Clin Invest. 2025 Jan 2;135(1):e163587. doi: 10.1172/JCI163587 (PMC11684799; doi:10.1172/JCI163587)
Supplement: Supplemental data [file jci-135-163587-s236.pdf]

SUPPLEMENTARY FIGURES

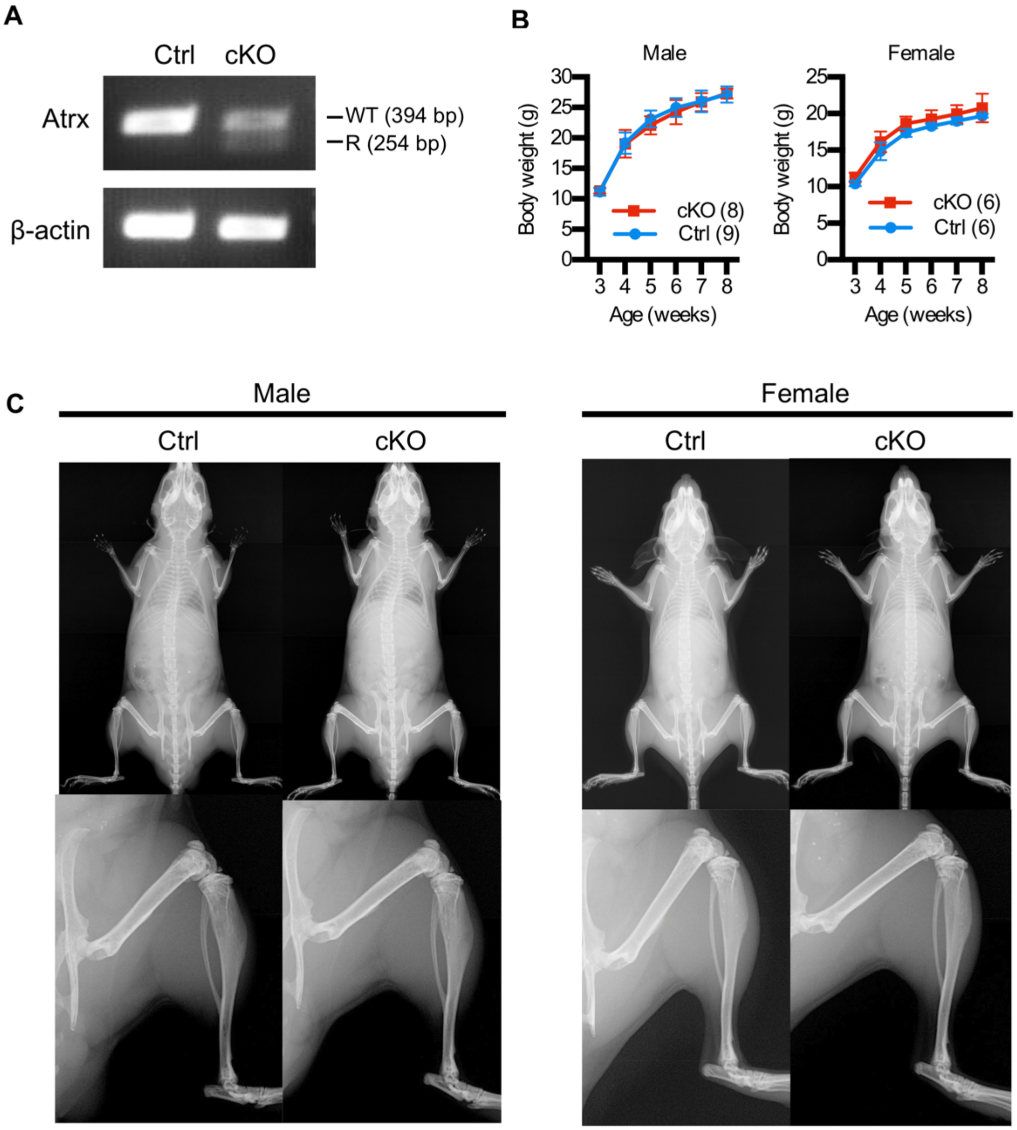

Fig. S1. Mice with specific *Atrx* deletion in pre-osteoblasts show decreased *Atrx* expression in bone but normal body weight and skeletal patterning. (A) RT–PCR analysis of RNA isolated from tibias of *Osterix-Cre; Atrx*$^{f/y}$ (cKO) and *Atrx*$^{f/y}$ control littermates (Ctrl). Primers were designed to flank the *loxP* sites in intron 17 and intron 20. Atrx wild-type transcript (WT) sizes as 394 bp while the Atrx recombinant one (R) as 254 bp. **(B)** Body weight analysis from 3 to 8 weeks of age in Atrx cKO and control male (n=8 for cKO; n=9 for Ctrl) and female (n=6 per

group) mice. Data are presented as means with ±SD. **(C)** Representative radiography images of 8-week-old Atrx cKO and control mice.

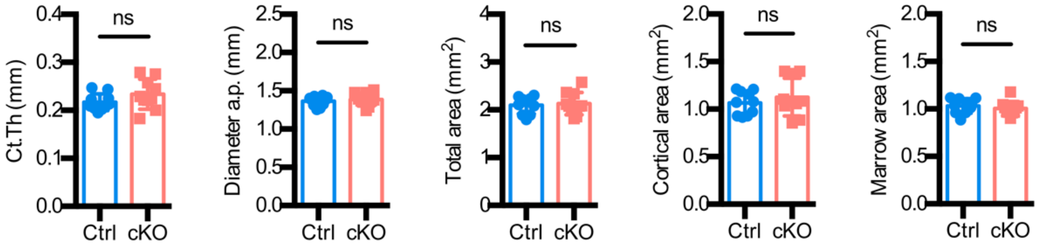

Figure S2. Cortical bone measurements in 8-week-old control and Atrx cKO male mice. Parameters of cortical microarchitecture were analyzed in femurs of 8-weelk-old male mice. n=10 per group. Data are presented as means with ±SD. Student *t* test, ns, not significant (P>0.05). Ct.Th, cortical thickness; Diameter a.p., anterior-posterior diameter; Total area, total cross-sectional area of cortical bone; Cortical area, cortical bone area; Marrow area, cortical marrow area.

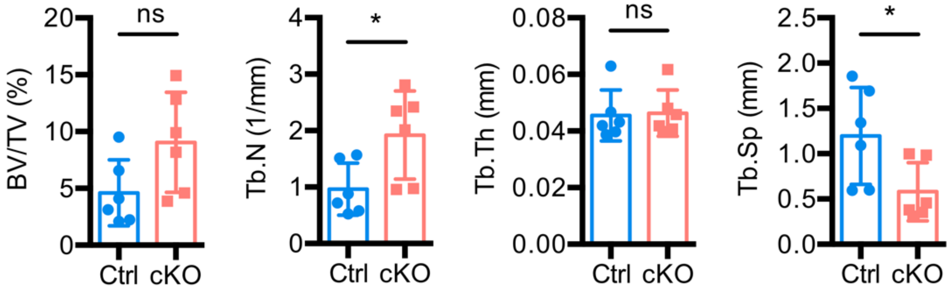

Fig. S3. 1.5-year-old Atrx cKO mice show increased trabecular bone mass. Parameters of trabecular microarchitecture (BV/TV, Tb.N, Tb.Th, Tb.Sp) were analyzed in femurs of 1.5-yeark-old male mice. cKO, *Osterix-Cre; Atrx*$^{f/y}$; Ctrl, *Atrx*$^{f/y}$ control littermates. n=6 per group. Data are presented as means with ±SD. Student *t* test, **P*<0.05. ns, not significant (*P*>0.05).

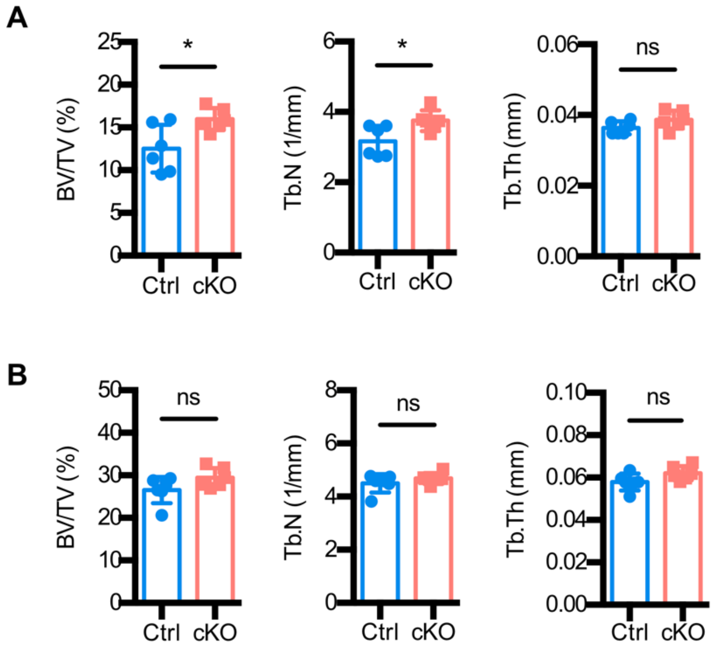

Fig. S4. 8-week-old Atrx cKO female mice show increased trabecular bone mass in femurs but not vertebrae. Parameters of trabecular (BV/TV, Tb.N, Tb.Th,) were analyzed in (A) femurs and (B) vertebrae of 8-week-old female mice. n=6 per group. Data are presented as means with ±SD. Student *t* test, *P<0.05; ns, not significant (P>0.05).

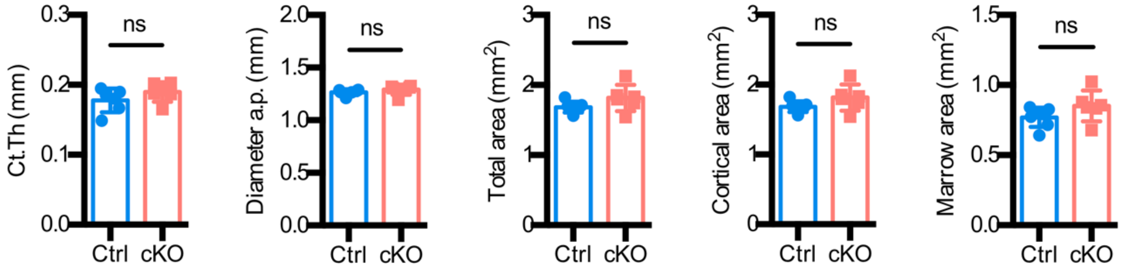

Fig. S5. Cortical bone measurements in 8-week-old control and Atrx cKO female mice.

Parameters of cortical microarchitecture were analyzed in femurs of 8-weelk-old female mice. n=6 per group. Data are presented as means with ±SD. Student *t* test, ns, not significant (P>0.05). Ct.Th, cortical thickness; Diameter a.p., anterior-posterior diameter; Total area, total cross-sectional area of cortical bone; Cortical area, cortical bone area; Marrow area, cortical marrow area.

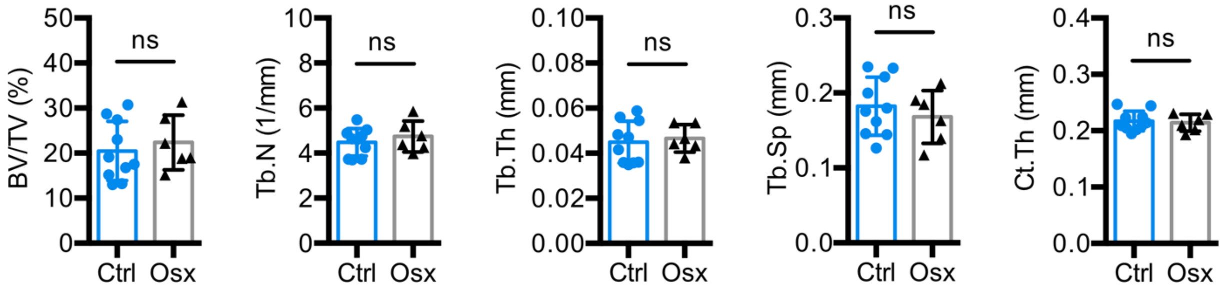

Fig. S6. *Osterix-Cre* (Osx) mice show similar bone phenotypes with *Atrx*$^{f/y}$ control mice. Parameters of trabecular microarchitecture (BV/TV, Tb.N, Tb.Th, Tb.Sp) were analyzed in femurs of 8-weelk-old male mice. n=10 for the control group; n=6 for the Osx group. Data are presented as means with ±SD. Student *t* test, ns, not significant (*P*>0.05).

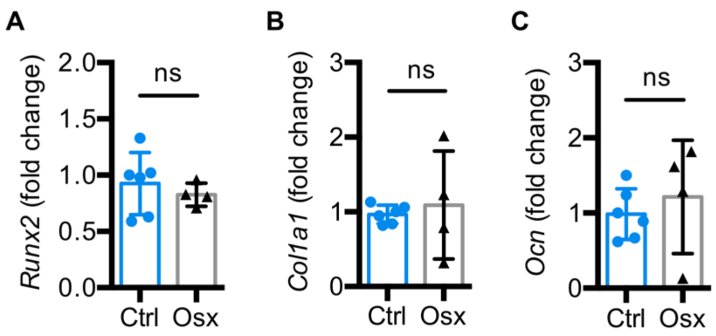

Fig. S7. Expression of osteoblast markers in control and Osx mice. (A-C) RT-qPCR results of *Runx2* (A), *Col1a1* (B), and *Ocn* (C) in control and Osx mice at 8 weeks of age. n=6 for the control group; n=4 for the Osx group. Data are presented as means with ±SD. Student *t* test, ns, not significant (P>0.05).

A

[GO analysis: upregulated genes]

Index	Name	P-value	Adjusted p-value
1	negative regulation of bone resorption (GO:0045779)	0.002997	0.04060
2	positive regulation of blood pressure (GO:0045777)	0.002997	0.04060
3	positive regulation of catecholamine secretion (GO:0033605)	0.002997	0.04060
4	positive regulation of neurological system process (GO:0031646)	0.003495	0.04060
5	negative regulation of appetite (GO:0032099)	0.003994	0.04060

B

[GO analysis: downregulated genes]

Index	Name	P-value	Adjusted p-value
1	striated muscle hypertrophy (GO:0014897)	0.003894	0.04497
2	negative regulation of osteoblast proliferation (GO:0033689)	0.004542	0.04497
3	positive regulation of membrane depolarization (GO:1904181)	0.004542	0.04497
4	neurotransmitter receptor transport, postsynaptic endosome to lysosome (GO:0098943)	0.004542	0.04497
5	postsynaptic neurotransmitter receptor diffusion trapping (GO:0098970)	0.005189	0.04497

Fig. S8. Gene ontology (GO) analysis of the differentially expressed genes from tibial RNA sequencing. (A-B) Gene ontology (biological process) analysis of the upregulated (A) and the downregulated gene sets (B).

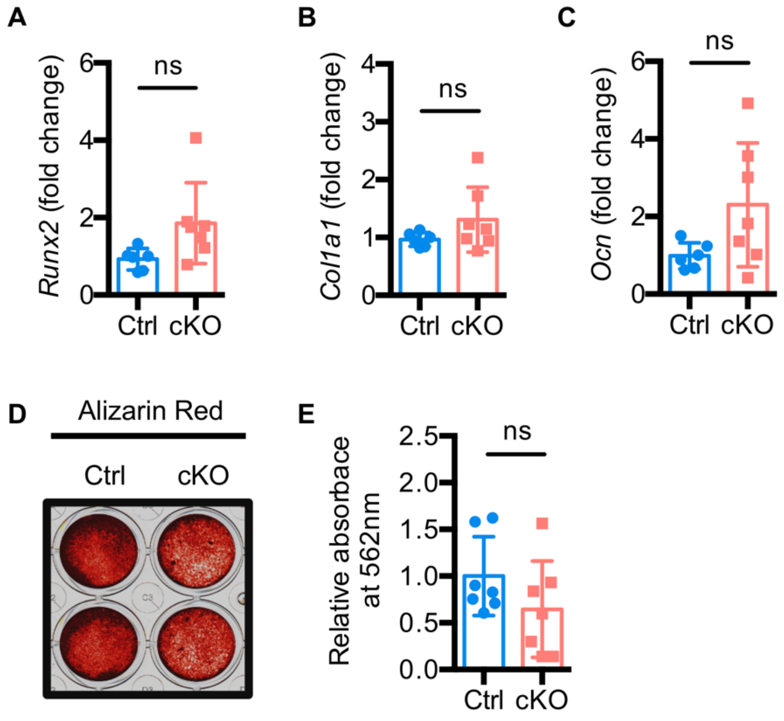

Fig. S9. Analysis of osteoblast markers expression and mineralization assays. (A-C) RT-qPCR results of *Runx2* (A), *Col1a1* (B), and *Ocn* (C) in tibias from control and Atrx cKO mice at 8 weeks of age. n=6 for $Atrx^{f/y}$ control; n=7 for Atrx cKO. **(D-E)** Alizarin Red S staining for evaluation of mineralization. (D), representative microscopic view of the cells after Alizarin Red S staining; (E), quantification of relative absorbance at 562 nm. n=7 per group. Data are presented as means with ±SD. Student *t* test, ns, not significant (P>0.05).

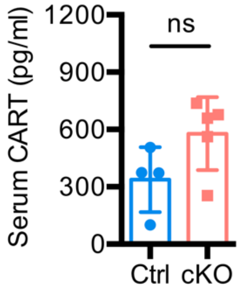

Fig. S10. Serum CART peptides levels in 1.5-year-old Atrx cKO male mice. ELISA of serum CART peptides at 1.5 years of age. n=4 for *Atrx*$^{f/y}$ control; n=6 for Atrx cKO. Data are presented as means with ±SD. Student *t* test, ns, not significant (P>0.05).

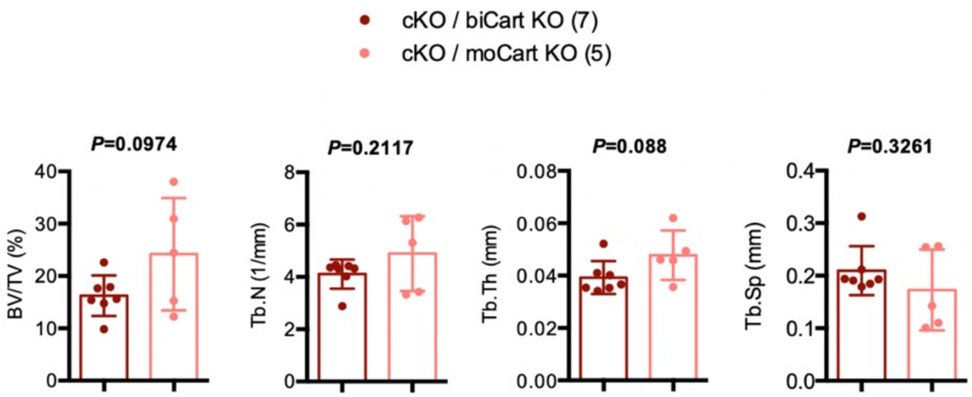

Figure S11. Trabecular bone measurements in *Cartpt/Atrx* double mutant founder mice. Biallelic or monoallelic *Cartpt* knockout mice were generated by CRISPR-Cas9 gene targeting (guide sequence: CTCGTGGGACGCATCATCCA) in the background of *Atrx* deletion (cKO). Parameters of trabecular (BV/TV, Tb.N, Tb.Th,) were analyzed in femurs of 8-week-old biallelic (n=7) or monoallelic (n=5) *Cartpt* knockout founder mice. Data are presented as means with ±SD. cKO, conditional *Atrx* deletion driven by *Osx-Cre*; biCart KO, biallelic *Cartpt* knockout; moCart KO, monoallelic *Cartpt* knockout.

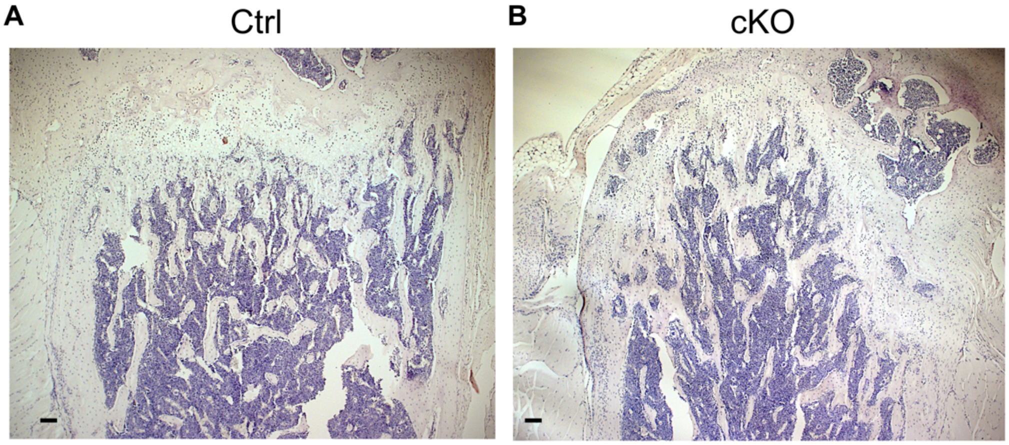

Fig. S12. Secondary antibody controls of IHC staining. (A-B) Anti-rabbit secondary antibody was applied in femur sections of control (A) and Atrx cKO (B) without overnight incubation of rabbit anti-CART peptides. Scale bar, 100 µm.

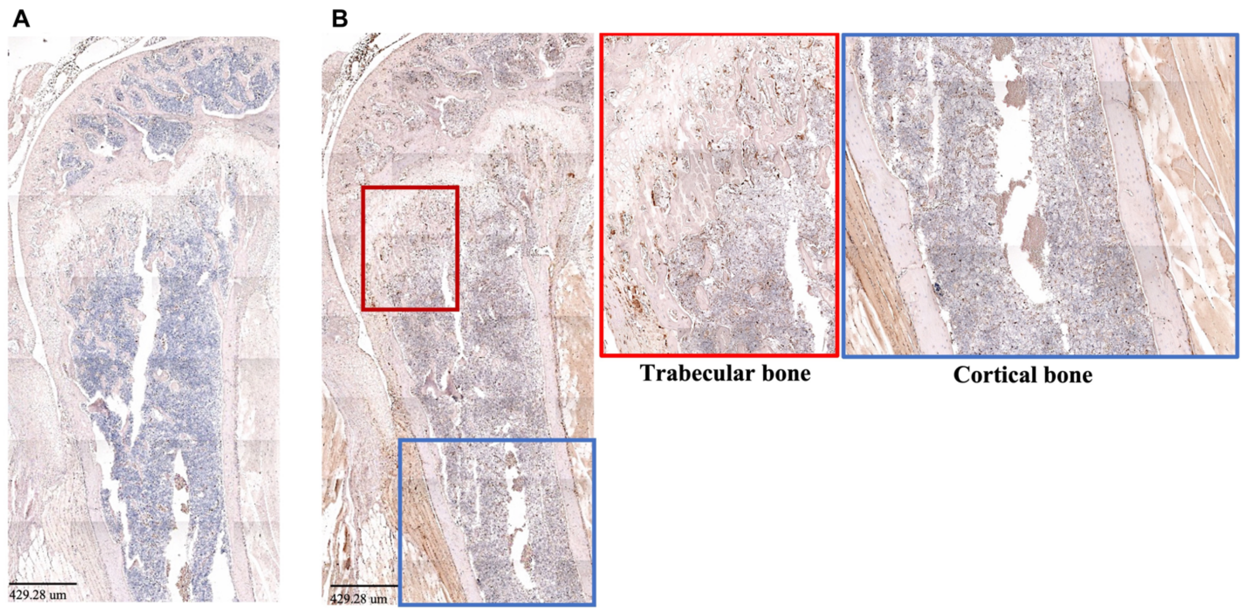

Fig. S13. ATRX is expressed in trabecular and cortical bone compartments in *Atrx*$^{f/y}$ mice. (A-B) Secondary antibody control (A) and ATRX IHC staining in trabecular (red square) and cortical bone (blue square) compartments in *Atrx*$^{f/y}$ mice. Representative images of ATRX IHC staining (n=3). Scale bar, 429.28 µm.

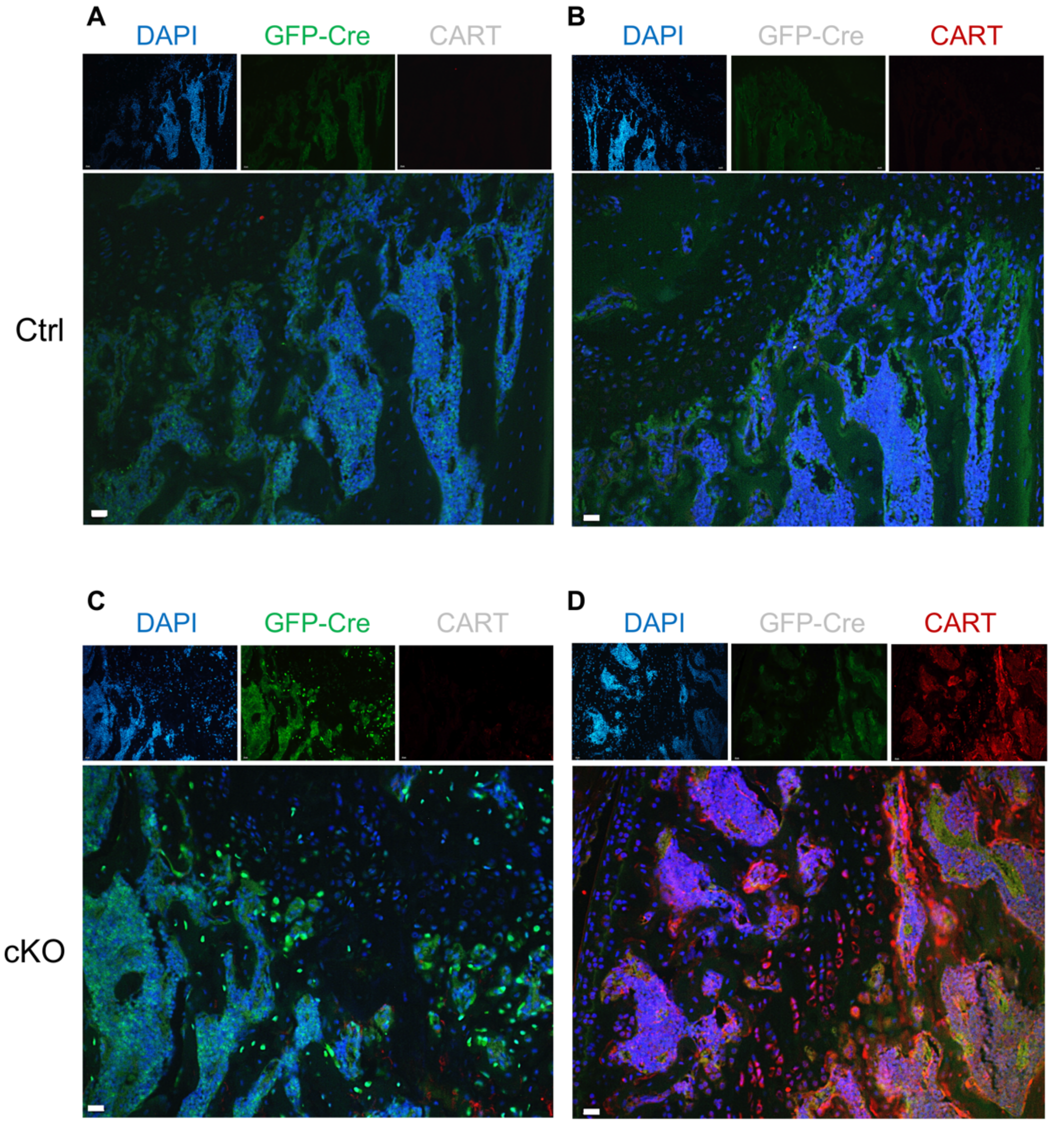

Fig. S14. Secondary antibody controls of IF staining. (A-B) The secondary antibody controls to anti-CART peptides (A) and anti-GFP (B) at 8 weeks of age in the control (*Atrx$^{f/y}$*) group. Scale bar, 20 µm. **(C-D)** The secondary antibody controls to anti-CART peptides (C) and anti-GFP (D) at 8 weeks of age in the Atrx cKO group. Scale bar, 20 µm.

Supplementary Table S1 - Expression level of *Atrx* and *Cartpt* in MC3T3

Gene Name	Cq-1	Cq-2	Cq-3	Cq MEAN
**Atrx**	23.66	23.32	23.33	23.44
**Cartpt**	33.42	33.82	33.71	33.65
**B2mg**	19.66	19.59	20.1	19.78

* ***Cq1-3*** *: 3 biological replicates of Cq value for each gene*

Supplementary Table S2 - List of oligonucleotides for genotyping, RT-PCR, RT-qPCR, ChIP- qPCR and prime editing

List of primers for genotyping (5'-3')		References
**Atrx**		*Nat Commun.* 2018 Mar 13;9(1):1057.
Atrx_forward	GGTTTTAGATGAAAATGAAGAG	
Atrx_reverse 1	TGAACCTGGGGACTTCTTTG	
Atrx_reverse 2	CCACCATGATATTCGGCAAG	
**Cre**		The JAX Lab
30295_Cre_F	GAGAATAGGAACTTCGGAATAGTAAC	
30296_Cre_R	CCCTGGAAGTGACTAGCATTG	

List of primers for RT-PCR (5'-3')		
**Atrx**		*PLoS One* . 2009 Sep 23;4(9):e7106.
17F	AGAACCGTTAGTGCAGGTTCA	
20R	ACCACCATCTTCTTGCCATC	
**Beta-actin**		
BA_F	TGGCGTGAGGGAGAGCATAG	
BA_R	GGGTGTGATGGTGGGAATGGGT	

List of primers for RT-qPCR (5'-3')	
Runx2 193_F	TCAGCAAAACTTCTTTTGGGAT
Runx2 193_R	CGGGCTCACGTCGCTCATC
Col1a1 4346_F	TTGGGGCAAGACAGTCATCGAAT
Col1a1 4346_R	TTGGGGTGGAGGGAGTTTACACGAA
Ocn_F	ACCCTGGCTGCGCTCTGTCTCT
Ocn_R	GATGCGTTTGTAGGCGGTCTTCA
Rankl_ZJ_F	GCTGGGCCAAGATCTCTAAC
Rankl_ZJ_R	GTAGGTACGCTTCCCGATGT
Opg 2108_F	CCAACAGTTTATCCAGCTGTCATGTC
Opg 2108_R	CCATATTCAAAGAGATCCAATGGAGTCTA
Atrx 358_F	TTGCCAAAAGGTACAGTGATTGT
Atrx 358_R	CGCGTTTTATGAGATTGTCAGC
Cartpt 192_F	GCGCTATGTTGCAGATCGAA
Cartpt 192_R	CTGTCGTCCCTTCACAAGCA
B2mg_F	GGTCTTTCTGGTGCTTGTC
B2mg_R	CGTATGTATCAGTCTCAGT

List of primers for ChIP-qPCR (5'-3')		
**RUNX2-ChIP**		
Smad4 intron_F	GCAAGCACACACACACATCA	*Genome Biol.* 2014 Mar 21;15(3):R52.
Smad4 intron_R	CTATTCTGGGCCTTGGTGGG	
Ocn promoter_F	TGGGGGTCATGTGCTCAGTGG	*Genome Biol.* 2014 Mar 21;15(3):R52.
Ocn promoter_R	CCAGAGCCTTGCCCAGGCAG	
Runx2 ChIP_128_F	GCAGCTGTATGTATCACTCACT	
Runx2 ChIP_128_R	CACCTCACACTGCTGTCTATC	
Runx2 ChIP_693_F	AAAGTGTGTAGAAGGAGAGCTAAA	
Runx2 ChIP_693_R	GAACCACTAAGCGAAGCTATCA	
**ATRX-ChIP**		
Rhbdf1-intron 6_F	ACTCAGGCTCTGTCTCTCAA	
Rhbdf1-intron 6_R	CTACTTTGATGCCCTCCTACAG	
mTel 2_F	CAAGTTTAAGGTTGTGTTTGTCAC	*Nat Commun.* 2018 Mar 13;9(1):1057.
mTel 2_R	AAATGAGTTGCAACAGGTACAAT	
mTel X_F	TGTCTGGGTCTTTGGAGAGG	*Nat Commun.* 2018 Mar 13;9(1):1057.
mTel X_R	CCTAACCCATCTGCTGGTTC	
Atrx ChIP_41_F	AAATACTGACCAGCTCCTTCTC	
Atrx ChIP_41_R	TGCTACTGCTACCTTTGCTG	
Atrx ChIP_142_F	AGCAAAGGTAGCAGTAGCAG	
Atrx ChIP_142_R	GCTATAAGAAGCCGGAGAGC	

Sequences of the pegRNA (5'-3')

Spacer oligo top	caccGTTTGCTGGGTGCCCGTGCCCgtttt
Spacer oligo bottom	ctctaaaacGGGCACGGGCACCCAGCAAAC
Extension oligo top	gtgcGCTCGGCGTCCTCCTGctaGGCACGGGCACC
Extension oligo bottom	aaaaGGTGCCCGTGCCtagCAGGAGGACGCCGAGC
Scaffold type1 oligo top	agagCTAGAAATAGCAAGTTAAAATAAGGCTAGTCCGTTATCAACTTGAAAAAGTGGCACCGAGTCG
Scaffold type1 oligo bottom	gcacCGACTCGGTGCCACTTTTTCAAGTTGATAACGGACTAGCCTTATTTTAACTTGCTATTTCTAG

Supplementary Table S3 - List of 23 differentially expressed genes
Upregulated DEGs (Log2-fold change ≥ 1; *P*-adjust < 0.05)

	Gene	Log2-fold change	*P*-adjust
ENSMUSG00000021647	Cartpt	9.577292082	0.000154
ENSMUSG00000038932	Tcfl5	3.954752818	1.68E-34
ENSMUSG00000094102	Ighv9-2	3.708275923	0.002106
ENSMUSG00000027577	Chrna4	3.42872236	0.002106
ENSMUSG00000050423	Ppp1r3g	3.426104625	0.002818
ENSMUSG00000040026	Saa3	2.253839843	0.000755
ENSMUSG00000046573	Lyrm4	1.830091876	1.68E-34
ENSMUSG00000076563	Igkv5-48	1.826736096	0.008979
ENSMUSG00000076564	Igkv12-46	1.816699769	0.005263
ENSMUSG00000074489	Bglap3	1.384044328	1.78E-06

Downregulated DEGs (Log2-fold change < 1; P-adjust < 0.05)

	Gene	Log2-fold change	*P*-adjust
ENSMUSG00000033578	Tmem35a	-1.057111483	0.040685
ENSMUSG00000044288	Cnr1	-1.170791094	0.001064
ENSMUSG00000021303	Gng4	-1.263768879	0.002106
ENSMUSG00000069806	Cacng7	-1.445563962	0.008979
ENSMUSG00000055409	Nell1	-1.625869383	0.004448
ENSMUSG00000020607	Fam84a	-1.652797344	0.000606
ENSMUSG00000039385	Cdh6	-1.738867295	3.04E-09
ENSMUSG00000039257	Vstm2b	-2.054472369	0.021621
ENSMUSG00000055632	Hmcn2	-2.131144414	0.002106
ENSMUSG00000026697	Myoc	-2.358795885	0.002106
ENSMUSG00000068417	Pnp2	-2.796611985	0.001267
ENSMUSG00000038760	Trhr	-2.908903728	0.004448
ENSMUSG00000021638	Ocln	-2.930462087	0.013126
