# Supplementary figures and images for: ATRX silences Cartpt expression in osteoblastic cells during skeletal development
Source: J Clin Invest. 2025 Jan 2;135(1):e163587. doi: 10.1172/JCI163587 (PMC11684799; doi:10.1172/JCI163587)

**Figure S12. Full unedited gel for Figure S1.**

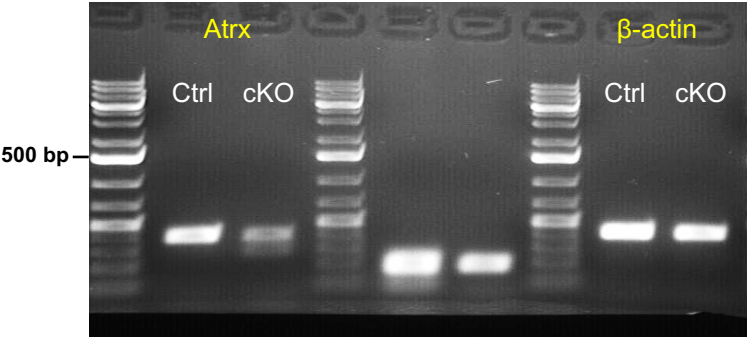

Supplement: Unedited blot and gel images [file jci-135-163587-s237.pdf]
